# Beliefs about accuracy shape confidence attributions to humans and artificial agents

**DOI:** 10.1038/s44271-026-00445-4

**Published:** 2026-04-22

**Authors:** Clara Colombatto, Stephen M. Fleming

**Affiliations:** 1 Department of Psychology, University of Waterloo, Waterloo, ON Canada; 2 Department of Experimental Psychology and Institute of Cognitive Neuroscience, University College London, London, UK; 3 Max Planck UCL Centre for Computational Psychiatry and Ageing Research, University College London, London, UK

**Keywords:** Human behaviour, Human behaviour

## Abstract

To effectively communicate and collaborate with others, we must monitor not only other people’s cognitive states (e.g., what someone thinks or believes) but also their metacognitive states (e.g., how confident they are in their beliefs). While humans routinely share confidence, either explicitly (e.g., “I am sure”) or implicitly (e.g., via response times), metacognitive capabilities are still developing in artificial intelligence (AI), raising the question of how humans attribute confidence to AI systems. In seven pre-registered experiments (post-exclusion *N*s = 113, 109, 56, 59, 52, 60, 57), participants observed human and AI agents make perceptual choices and reported how confident the observed agent seemed in each choice. Overall, attributions of confidence were sensitive to observed behaviour (e.g., task difficulty, accuracy, and response times), but also agent type: observers consistently overestimated the confidence of AI agents compared to humans—even when their behaviour was identical. This illusion of greater confidence in AI decisions was robust across behavioural profiles, agent descriptions, and decision-making domains (visual perception, general knowledge) but was reduced in more subjective decisions (emotion categorisation). An experimental manipulation further showed that illusions of confidence are rooted in prior beliefs about the agents’ capabilities. Together, these investigations of metacognitive attributions reveal a powerful illusion of confidence in artificial systems and highlight a central role for attributions of metacognitive states in human-human and human-AI interactions.

## Abstract (web summary)

This work shows how humans infer others’ confidence from their observed behaviour and prior beliefs about the agents’ accuracy. As a result, they overestimate the confidence of AI agents compared to other humans, even when behaviour is identical—an illusion of AI confidence.

## Introduction

Autonomous systems are increasingly pervasive in our daily lives—from personal assistance and product suggestions to healthcare recommendations and automated transportation. As artificial intelligence (AI) becomes progressively advanced and ubiquitous, key questions arise regarding how humans interact with machines. For example, building more optimal recommendation algorithms is useful only insofar as humans are willing to trust their suggestions, and designing more skilled robots is useful only insofar as humans are willing to rely on their assistance. Technological advances must thus be complemented with a psychology of human-machine collaboration—a novel challenge for cognitive science^1^.

The study of human-machine interactions is especially pressing given that they likely involve different processes compared to human-human interactions^2^. Recent work on human-computer interactions has shown that humans ascribe different abilities to artificial systems^3–7^. Even when algorithms perform equally well if not better than humans, users sometimes hesitate to rely on advice or help from algorithms—a phenomenon known as ‘algorithm aversion’ (refs. ^8,9^; for a review, see ref. ^10^; c.f. refs. ^4,6,11^). While attitudes towards AI can be enhanced by factors such as affective abilities^12^, physical presence^13^, or decision interpretability^14^, a prior belief that behaviour is generated by a computer (vs. a human) can be sufficient to influence perceptions of otherwise identical actions^15,16^.

One striking difference between human-human and human-machine interactions is that when we make decisions with other people, we have access not only to their behaviour but also to their metacognitive states—for instance, how confident they are in a belief or decision (for a review, see ref. ^17^). In humans, such confidence can be revealed not only explicitly, via verbal estimates^18,19^, but also implicitly, via response times^20,21^, movement dynamics^22,23^, and speech prosody^24,25^. In other words, humans regularly *infer* or *attribute* others’ confidence by tracking explicit and implicit signatures of decision confidence. This type of information is highly valuable in collaborative decision-making^26^: for example, it helps resolve disagreements among group members^27^, and indeed, group decisions are more effective than individual ones only when confidence estimates are shared^28^.

If attributions of confidence are key to human-human collaboration, they should also be central to human-machine interactions. However, little is currently known about how humans attribute confidence to AI systems. In fact, this type of inference is difficult when interacting with machines that are typically designed to fulfil first-order rather than metacognitive functions, and which vary in the extent to which they can provide calibrated estimates of uncertainty. Indeed, AI’s capacity for metacognition, including the ability to provide accurate confidence reports, is developing^29–33^, and current models sometimes express confidence estimates that do not track their underlying accuracy^34^. For example, while in humans confidence typically decreases as tasks get harder, some models show the opposite pattern^35^. Beyond assessments of actual metacognitive capacities, AI confidence estimates are rarely explicitly communicated to human collaborators (ref. ^36^; for reviews, see refs. ^37,38^). However, the format of their output often contains cues that, in humans, typically do signal confidence *implicitly*, such as system response times, which are akin to human response times. Together, these considerations raise foundational questions around how humans attribute confidence to AI systems when this is not communicated explicitly.

On the one hand, there is reason to suspect that attributions of confidence may differ between humans and AI on the basis of agents’ beliefs about their respective capabilities. For example, humans are typically more confident when they are more likely to be correct (i.e., they exhibit some degree of metacognitive calibration^39^). As a result, a tendency to overestimate AI’s capabilities may result in inflated attributions of confidence for AI. These same agent-specific properties also underlie the distinction between inferences of others’ confidence and participants’ own confidence in others’ choices (e.g. refs. ^40,41^), since an advisor may be confident in responses that an observer knows to be inaccurate, revealing a discrepancy between other- and self-directed metacognition.

But beyond general beliefs about agents’ abilities, inferences about confidence should also depend on momentary assessments of individual decisions. A large body of work in perceptual metacognition has shown how the same agent can be more or less confident depending on properties of specific decisions (e.g., difficulty), as well as their own performance on individual trials (e.g., accuracy and response times). In particular, confidence is typically higher when the evidence is easier to discriminate (i.e., on easier trials) and the agent is more likely to be accurate^39^. This relationship between difficulty and confidence, however, is disrupted when agents make errors, where they instead report lower confidence on easier trials—where, presumably, they become aware that they have made an error^39,42^. In summary, past work in perceptual metacognition has shown how confidence is shaped by both agent-specific properties and ongoing behaviours, raising fundamental questions about the relative role of prior beliefs and momentary observations in the attribution of confidence to other systems.

Here we set out to determine how both agent-specific beliefs and trial-specific performance govern attributions of confidence to humans and AI systems. Participants watched other agents make choices and were asked to determine how confident the agents were in their choices. This design allowed us to investigate how attributions of confidence are modulated by decision variables such as task difficulty, observed accuracy, and observed response time. At the same time, we kept these overall behaviours constant across different agents and varied the nature of the agents making the choices—which allowed us to examine how attributions of confidence may depend on prior beliefs about the agents, even when actual performance was in fact identical.

In additional experiments, we tested the robustness and generalisability of these results, examining how confidence is attributed when machines display human-like (Experiment 1) or stereotyped behaviour (Experiment 2), when artificial agents are described in more or less anthropomorphic ways (Experiment 3), and when the task involves a general knowledge quiz rather than perceptual judgments (Experiment 4). We also asked whether confidence attributions impacted participants’ willingness to take advice from AI systems (Experiment 5), and whether similar results held in a subjective task involving emotion categorisation (Experiment 6). In a final experiment, we tested a causal role for prior beliefs about the agents’ ability in attributions of confidence (Experiment 7). Throughout these experiments, we also measured global beliefs about the agents’ competence and trustworthiness, and investigated the relationship between inferred confidence and social impressions. Overall, these results characterise how people attribute confidence to both humans and machines, and how even illusory attributions of confidence influence perceived trustworthiness and collaborative behaviour in human-human and human-machine interactions.

## Methods

**Fig. 1:**
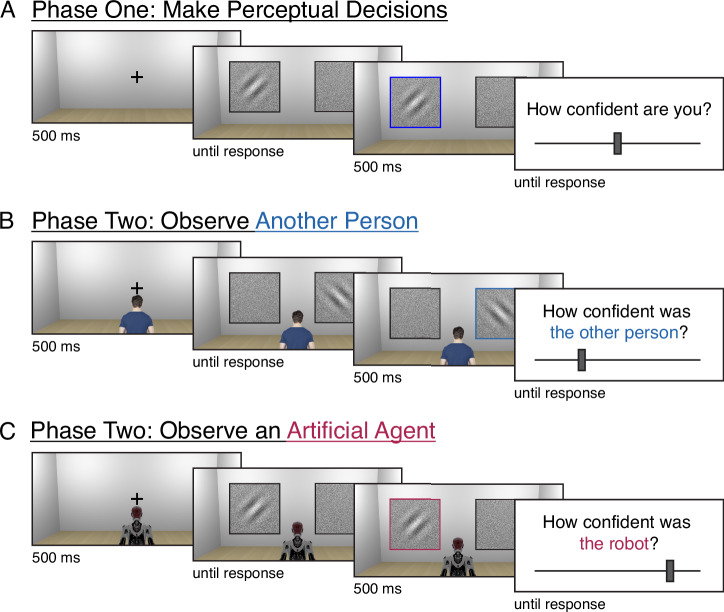
Overview of design for Experiment 1. **A** Participants first completed a perceptual decision-making task where they selected which of two stimuli contained a Gabor patch (Phase 1; ‘Self ’). **B**, **C** Next, participants observed other agents complete the same task, and reported their assessment of the other agent’s confidence (Phase 2; ‘Other’). The other agent was described and depicted as either another person (**B**) or a robot (**C**), although their actual performance on the task was identical.

To investigate how people attribute confidence to others, we designed a task divided into two phases, as illustrated in Fig. 1. In the first phase (‘Self’), participants made a series of perceptual decisions—namely, which of two noise patches contained a Gabor stimulus with varying opacity (Fig. 1A). In the second phase (‘Other’), participants watched other agents make these decisions, with these agents being either another person (Fig. 1B) or a robot (Fig. 1C), presented in a randomised order. Importantly, the same behavioural parameters and generative functions determined the behaviour of both the other person and the robot, such that within each participant, performance was equated across the two agents. We hypothesised that estimates of other agents’ confidence would depend on the difficulty of the task, but also on their behaviour (namely, accuracy and response times), as well as on their nature (human vs. artificial).

All experiments were pre-registered (Exp. 1: https://osf.io/um5h2/, preregistered on December 20, 2022; Exp. 2: https://osf.io/s8ebv/, preregistered on February 8, 2023; Exp. 3: https://osf.io/vue42/, preregistered on March 8, 2023; Exp. 4: https://osf.io/y3zu8/, preregistered on March 20, 2023; Exp. 5: https://osf.io/8ca53/, preregistered on April 26, 2023; Exp. 6: https://osf.io/mdntc/, preregistered on April 14, 2024; Exp. 7: https://osf.io/sderm/, preregistered on September 11, 2024). All methods and procedures were approved by the UCL Research Ethics Committee, and all participants provided their informed consent.

### Participants

For each experiment, participants were recruited via Prolific (prolific.co^43^) in exchange for monetary compensation. Participants were able to take part in the experiments if they were fluent in English, had a Prolific task approval rate of at least 95%, and had not previously participated in any of the other studies reported here. As per our pre-registered plan, we recruited *N* = 120 participants for Experiment 1. This sample size was determined via a power analysis of pilot data (*N* = 18) and was fixed for Experiment 2. The sample size for the remaining experiments was halved to *N* = 60 based on a power analysis of Experiments 1 and 2. In Experiment 7, we collected a sample of *N* = 60 as per our preregistered plan; however, we observed a higher exclusion rate for our manipulation check (see criterion #4 below), and so we recruited additional participants until *N* = 60 participants passed this check.

Participants were excluded according to our pre-registered plans if they (1) had a viewport size smaller than 720 × 500px (*N* = 1 in Experiment 4); (2) reported having encountered problems (see Debriefing; *N* = 2 in Experiment 1, *N* = 1 in Experiment 2); (3) failed to answer our open-ended text questions sensibly (see Debriefing; *N* = 1 in Experiment 1, *N* = 1 in Experiment 5); (4) had a mean accuracy lower than 70% in the catch questions about the agents’ confidence in Experiment 5 (*N* = 6), and about the agents’ accuracy in Phase 1 of Experiment 7 (*N* = 17); (5) converged on a target with opacity level lower than 5% in Phase 1 (*N* = 4, 10, 4, 1; in Experiments 1, 2, 3, and 5, respectively); and (6) provided uniform confidence ratings in Phase 2 (i.e. with 80% of ratings below 10% or above 90%; *N* = 3 in Experiment 7). No participants participated more than once as determined via their Prolific IDs. The final sample sizes were thus *N* = 113 in Experiment 1 (self-reported 26 females, 87 males; M_age_ = 31.65; data on race/ethnicity were not collected), *N* = 109 in Experiment 2 (32 females, 77 males; M_age_ = 33.76), *N* = 56 in Experiment 3 (25 females, 31 males; M_age_ = 33.07), *N* = 59 in Experiment 4 (24 females, 35 males; M_age_ = 33.22), *N* = 52 in Experiment 5 (21 females, 31 males; M_age_ = 33.71), *N* = 60 in Experiment 6 (25 females, 35 males; M_age_ = 31.12), and *N* = 57 in Experiment 7 (21 females, 35 males, 1 non-binary; M_age_ = 30.82).

### Apparatus

Participants completed the experiment on their own devices, and were redirected to a website hosted on the JATOS server^44^. Stimulus presentation and data collection were controlled via custom software written in HTML, CSS, JavaScript, and PHP, using the jsPsych library^45^. Their browser window was automatically put in full-screen mode at the beginning of the experiment.

### Experiments 1–3

#### Phase 1: Self

Participants began by completing an image detection task illustrated in Fig. 1A. On each trial, they were shown two noise images side by side, and one also contained a Gabor patch. The noise images consisted of randomly intermixed white and black pixels, and were randomly selected for each participant on each trial without replacement amongst 1200 possible images. The Gabor patches consisted of sinusoidal gratings tilted at an orientation of 45° or 135° and were superimposed over the noise with varying transparency, thus varying detection difficulty. Participants indicated which of the two images contained a stimulus via a keypress (‘f’ for left, ‘j’ for right), and then rated how confident they were in this choice.

This first phase began with some practice trials, where all gratings were presented at 100% opacity and feedback was given after each trial. Each trial in these practice blocks comprised: (1) a fixation cross (in 42px black Helvetica font), for 500 ms; (2) the stimuli (120 × 120px with a 3px black border each, centred on the screen vertically and at −140 and 140px horizontally), until response; and (3) response feedback (“Correct” or “Wrong”) and highlight (in green [#3CB371] if correct, red [#FF0000] if incorrect), for 1000 ms. Each block comprised 4 trials (2 grating orientations [45, 135 degrees] ×2 grating positions [left, right]) in a random order, and was repeated for each participant until their mean accuracy on the last block was at or above 75%.

Next, participants completed an experimental phase, where the grating opacity started at 100%, and was then updated on each block via a staircasing procedure: it decreased if accuracy in the last block was above 75%; increased if accuracy in the last block was below 60%; and remained unchanged otherwise. Decreases and increases occurred in steps of 50, 20, 10, 10, 5, 5, 2, 2, 2, and 1 until convergence—with a maximum and minimum opacity value of 100 and 0, respectively. Each trial comprised: (1) a fixation cross, for 500 ms; (2) the stimuli, until response; (3) a blue (#0000FF) response highlight, for 500 ms; and (4) a confidence prompt, until response (“How confident are you?”, to be answered with a slider from 0 [“I am not confident at all”] to 100 [“I am very confident”]). Each block comprised 8 trials (2 grating orientations [45, 135 degrees] × 2 grating positions [left, right] × 2 repetitions) in a random order, and was repeated for each participant for a maximum of 200 trials, or until (1) they completed at least 152 trials; and (2) their accuracy in the last 3 blocks was above 60% and below or equal to 75%.

#### Phase 2: Other

Participants were then introduced to the idea that people can differ quite a lot in how confident they are in their choices, and they were shown sample distributions of confidence ratings from several previous participants for the same choice difficulty. They then began a series of blocks where they were shown choices from other agents and guessed how confident the other agent was in that choice. The other agent was either another participant (Fig. 1B) or an artificial agent (Fig. 1C)—the key manipulation of interest. The artificial agent was referred to either as a ‘robot’ (Experiments 1 and 2) or as a ‘computer algorithm’ (Experiment 3).

Each trial in this phase comprised: (1) a fixation cross, for 500 ms; (2) the stimuli, until the other agents’ response time (see below); (3) a response highlight, for 500 ms; and (4) a confidence prompt, until response (“How confident do you think the [other person/robot/computer algorithm] was?”, to be answered with a slider from 0 [“The other person/robot/computer algorithm was not confident at all”] to 100 [“The other person/robot/computer algorithm was very confident”]). The opacity of the Gabor stimulus was varied from −5% to 5%, with 0% being the optimal difficulty level determined in Phase 1—with a maximum and minimum opacity value of 100 and 0, respectively.

Each observer saw two blocks from each agent in a randomised order (i.e., two ‘other person’ blocks followed by two ‘robot/computer algorithm’ blocks, or vice versa), and each block comprised 44 trials (2 grating orientations [45, 135 degrees] × 2 grating positions [left, right] × 11 difficulty levels [from −5 to +5]) in a randomised order. To remind participants of which agent they were observing, we randomly assigned a colour cue (blue [#3C3CA3] or purple [#841F42]) to each agent, separately for each participant. In Experiments 1 and 2, the agent was also depicted in the centre of the screen from the back, as if they were observing the stimuli. Participants were instructed that the image chosen by the other agent would be highlighted at the same time the other person made their choice. Every 11 trials (except for the last trial of each block pair), participants were given feedback about their cumulative performance on this confidence estimation task (as the absolute difference between the estimated confidence and the agents’ confidence [see below]).

Both Phase 1 and the two halves of Phase 2 were preceded by comprehension questions regarding which question participants would be asked in the trials to follow (e.g., “After observing the other participant’s choice, you will be asked a question. Which one?”).

#### Observed behaviour

In Experiment 1, the agents’ behaviour was determined by matching each observer with one of 20 counterpart participants who had previously completed a similar version of the experiment. In particular, these participants had completed a staircasing procedure (similar to Phase 1 as described above), followed by four blocks of experimental trials of the Gabor discrimination task, with 44 trials each (2 grating orientations [45, 135 degrees] × 2 grating positions [left, right] × 11 difficulty levels [from −5 to +5]).

These data were then used to generate the trials for Experiment 1, where on each trial we determined (1) the agent’s accuracy, computed from the probability that the counterpart participant would respond correctly at that difficulty level based on a psychometric function fit to their performance; (2) the agent’s response time, randomly picked from a gaussian distribution with mean and standard deviation based on the counterpart participant’s response times at that difficulty level and accuracy (but only including trials with response time between 200 ms and 3000 ms, and with these randomly picked values being replaced if they were lower than 200 ms); and (3) the agent’s confidence, randomly picked from a gaussian distribution with mean and standard deviation based on the counterpart participant’s confidence at that difficulty level and accuracy. If the counterpart participant had no trials corresponding to that difficulty level and accuracy, we determined (2) and (3) based on all other trials. Crucially, these generative functions were kept constant for each participant across both agents.

In Experiments 2 and 3, the agents’ behaviour was determined not from counterpart participants, but rather from an algorithm. This was a neural network implemented in TensorFlow^46^ with three convolutional blocks, each with a max pooling layer, and a fully-connected layer on top. The model was trained to discriminate between Gabor patches and noise stimuli, and an optimal opacity level was selected such that the model would have an average accuracy between 60 and 75% (thus matching the staircasing procedure from Experiment 1). On each trial we determined (1) the agent’s accuracy, computed from the probability that the model would correctly classify a stimulus at that subjective difficulty level based on a psychometric function fit to its performance; (2) the agent’s response time, randomly picked from a gaussian distribution with a mean of 250 ms and a standard deviation of 20 ms; and (3) the agent’s confidence, randomly picked from a gaussian distribution with mean and standard deviation based on the model’s mean accuracy at that difficulty level. Again, these generative functions were importantly kept constant for each participant across both agents.

#### Debriefing

At the end of the experiment, participants were asked a series of questions regarding their performance (i.e., “How well do you think you did at deciding which box contained the grating?”, and “How well do you think you did at guessing the [other participant/robot/computer algorithm]’s confidence?”), the other agents’ performance (i.e., “How well do you think the [other participant/robot/computer algorithm] did on this task?”), the other agents’ trustworthiness (i.e., “How much would you trust the [other participant/robot/computer algorithm] if you were working together?”), the artificial agent’s functionality (“How do you think the robot/computer algorithm functions? What do you think its capabilities are?”). They were also asked about their demographics (age and gender) and experience in the survey (e.g. technical difficulties or interruptions).

### Experiment 4

In this experiment, participants watched other agents select which of two countries had a larger (or smaller) population and later rated how confident they thought the other agent was in that choice. Each trial thus comprised: (1) a question (“Which of the two countries has a larger/smaller population?”), for 500 ms; (2) the question and response options, until the other agents’ response time (see below); (3) a response highlight, for 500 ms; and (4) a confidence prompt, until response.

Each participant completed a total of 176 trials divided into two blocks—one for the other person (“another participant who previously completed this task”), and one for the computer algorithm (“a computer algorithm that was built and trained to complete this task”) in a randomised order, with the respective colour cues also randomised. The debriefing questions were the same as in previous experiments, except participants were no longer asked about their own performance in the task.

#### Observed behaviour

The trial characteristics and agents’ behaviour were determined by matching each observer with one of 10 counterpart participants who had previously completed the general knowledge task. For this task, we first selected the 53 countries with populations between 25 and 500 million based on the 2021 UN census (https://population.un.org/wpp/). For each counterpart participant, we then randomly selected 176 pairs of countries out of all possible combinations and asked them to select the one with the smaller or larger population—resulting in 176 trials (2 question framings [“smaller”, “larger”] × 2 country positions [smaller, larger on the left] × 44 country pairs). Each participant in Experiment 4 was then paired with one of these 10 counterpart participants and saw all their 176 trials in a random order, each randomly assigned to either the ‘other person’ or the ‘computer algorithm’ block. The trial characteristics (i.e., prompt and response options) and observed behaviour (i.e., accuracy, response time, and confidence) thus matched the counterpart participants’ behaviour exactly—with the only constraint being a maximum response time of 12 s.

### Experiment 5

In this experiment, participants received advice from other agents in a perceptual decision-making task. Phase 1 was identical to Experiments 1–3, except participants were no longer asked about their own confidence. Phase 2 was instead adapted such that participants first made their own judgments, and later received the advice of another agent, after which they had the opportunity to revise their initial choices. Each trial thus comprised: (1) a fixation cross, for 500 ms; (2) the stimuli, until response; (3) a response highlight, for 500 ms; and (4) a screen with the advice (e.g., “Your choice: right; Other participant/Computer algorithm’s choice: left”) and reversal prompt (“Would you like to change your initial choice? Press ‘y’ for yes, ‘n’ for no.”), until response. On some trials (with 10% probability each), this was followed by (5) a catch question (“What was the [other participant/computer algorithm]’s advice on the last trial?”), until response; and (6) feedback on this catch question (“Correct!” or “Wrong!”), for 500 ms.

Each participant completed four blocks of 44 trials each (2 grating orientations [45, 135 degrees] × 2 grating positions [left, right] × 11 difficulty levels [from −5 to +5]) in a randomised order. Two of the four blocks had advice from another person (“another participant who previously completed this task”), and two had advice from the computer algorithm (“a computer algorithm that was built and trained to complete this task”), with the respective colour cues also randomised. The accuracy of the agents was determined as in Experiment 1, and every 11 trials (except for the last of each block pair), participants received feedback on their accuracy based on their revised choices. The debriefing questions were the same as in previous experiments, except participants were no longer asked about their accuracy in guessing the other agents’ confidence.

### Experiment 6

In this experiment, participants watched other agents select which of six emotions a face seemed to display and later rated how confident they thought the other agent was in that choice. Each trial thus comprised: (1) a question (“Which emotion is this face displaying?”), for 500 ms; (2) the question, the face, and the response options, until the other agents’ response time (see below); (3) a response highlight, for 500 ms; and (4) a confidence prompt, until response. Each participant completed a total of 180 trials divided into two blocks—one for the other person, and one for the computer algorithm, in a randomised order. The experiment was otherwise identical to previous ones.

#### Observed behaviour

The trial characteristics and agents’ behaviour were determined by matching each observer with one of 10 counterpart participants who had previously completed the emotion categorisation task. Each counterpart participant saw a total of 180 faces (30 identities × 6 emotions [Afraid, Angry, Disgusted, Happy, Sad, Surprised]). The faces were drawn from the Karolinska Directed Emotional Faces dataset^47^, and were shown to each counterpart participant in a random order. Each participant in Experiment 6 was then paired with one of these 10 counterpart participants and saw all their 180 trials in a random order, each randomly assigned to either the ‘other person’ or the ‘computer algorithm’ block. The trial characteristics (i.e., stimulus) and observed behaviour (i.e., choice) thus matched the counterpart participants’ behaviour exactly—with the only constraint being a maximum response time of 12 s.

### Experiment 7

This experiment was divided into two parts. In Phase 1, participants observed choices of two algorithms that varied in their accuracy. In Phase 2, they were then asked to report their perceived confidence on trials in which the same two algorithms were adjusted to have equal accuracy (Phase 2). The choices involved the same perceptual decision-making task as in experiments 1–3, and so the two phases were preceded by the same instructions and practice phase, which was repeated until participants achieved at least 75% accuracy on the last block (see Experiments 1–3: Self).

#### Phase 1: Observation

Participants were told that in the experiment, they would be observing two computer algorithms labelled ‘Algorithm A’ and ‘Algorithm B’. They then completed two blocks of observation trials, where they were shown the choices of the two Algorithms, one at a time and in a randomised order, and with their respective colour cues [blue/purple] also randomised. Each trial in this phase comprised: (1) a fixation cross, for 500 ms; (2) the stimuli, until the other agents’ response time (see below); and (3) a response highlight with feedback, for 500 ms. To ensure attention, on a subset of trials (i.e., with 10% probability on each trial), the feedback screen was followed by an attention check, where participants were asked to report the algorithm’s performance on the last trial (correct or incorrect). The opacity of the Gabor stimulus was varied from −5% to 5%, with 0% being the optimal difficulty level derived from the median of participants in Experiment 1 (8%). Each block comprised a total of 44 trials (2 grating orientations [45, 135 degrees] × 2 grating positions [left, right] × 11 difficulty levels [from −5 to +5]) displayed in a randomised order.

The choices of the algorithms were determined based on a cumulative Gaussian distribution, with the mean and standard deviation based on a neural network trained to achieve 70% performance (see Experiments 2–3). Critically, the behaviour of the two Algorithms was manipulated by shifting the mean accuracy by 30%, resulting in an overall mean accuracy of 54% for the ‘low accuracy prior’ algorithm, and 79% for the ‘high accuracy prior’ algorithm. The response times of the agents was randomly selected from a Gaussian distribution with a mean of 250 ms and a standard deviation of 20 ms, as in previous experiments.

#### Phase 2: Inferences

Participants were then informed that they would again observe the algorithm’s choices, but they would now also be asked to report how confident the algorithm seemed in that choice. They then completed two blocks of experimental trials, with inferences about each Algorithm interleaved in a random order throughout each block. Each trial in this phase comprised: (1) a fixation cross, for 500 ms; (2) the stimuli, until the other agents’ response time; (3) a response highlight (with no feedback), for 500 ms; and (4) a confidence prompt, until response (“How confident do you think algorithm A was?” or “How confident do you think algorithm B was?”, to be answered with a slider from 0 [“Algorithm [A/B] was not confident at all”] to 100 [“Algorithm [A/B] was very confident”]). As in Phase 1, each block comprised a total of 44 trials (2 grating orientations [45, 135 degrees] × 2 grating positions [left, right] × 11 difficulty levels [from −5 to +5]) displayed in a randomised order.

The choices of the algorithms were determined as in Phase 1, although importantly, the accuracy of the algorithms was no longer manipulated, and was identical for the two algorithms (mean = 65%).

### Statistical analysis

All analyses were conducted using R (R Core Team, 2020), RStudio (Rstudio Team, 2020), and packages lme4^48^, lmerTest^49^, and emmeans^50^.

To examine the impact of observed performance on perceived confidence, we conducted linear mixed-effects models of the effect of task difficulty (target opacity from −5 to +5 [Experiments 1–3, 7] or log ratio of the populations of the two countries [Experiment 4]; not included in Experiment 6), accuracy (coded as error: −0.5, correct: 0.5), and response times (Experiments 1, 4, and 6 only) on the confidence estimates, with counterpart participant (out of 20 participants in Experiments 1 and 5; out of 10 participants in Experiments 4 and 6) and observer number as random intercepts. To examine the relationship between self-directed metacognition and perceived metacognition, we first quantified the effect of trial difficulty on confidence attributions for each observer, agent, and accuracy, by fitting separate linear regression models predicting confidence estimates from difficulty. From these coefficients, we then operationalised other-directed metacognition as the difference between slopes on accurate vs. inaccurate trials, separately for each subject and agent. Self-directed metacognition was operationalised as metacognitive efficiency computed via a hierarchical regression model (the RHmeta-d model, an extended version of the HMeta-d model; see https://github.com/metacoglab/HMeta-d^51^).

To test whether attributions of confidence might vary depending on whether they concern another person as opposed to an artificial agent, we added the agent to the first model (along with accuracy, response times, and difficulty [omitted in Experiment 6]) as a fixed factor (coded as other person: −0.5, robot [Experiments 1–2] or algorithm [Experiments 3–4, 6]: 0.5; and in Experiment 7 low accuracy prior: −0.5, high accuracy prior: 0.5). To further deconstruct this effect, in Experiment 1 we ran an exploratory analysis predicting attributions of confidence from accuracy (interacting with difficulty), response time, and agent on the current trial, as well as accuracy (interacting with difficulty), response time, and confidence on each of the previous five trials, again with observer number as a random intercept, and discarding the first 5 trials of each block. Next, we checked the consistency of this model across agents by adding agent as an interactive factor to all terms.

To examine learning effects, we conducted linear mixed-effects models of the effect of trial epoch (in 11 intervals of 8 trials each in Experiments 1–4 and 7, and 10 intervals of 9 trials each in Experiment 6), agent (robot vs. person; low vs. high accuracy prior in Experiment 7), and block order (robot first vs. person first; low vs. high accuracy prior first in Experiment 7) on the confidence estimates, with observer number as a random intercept.

To examine advice-taking behaviour in Experiment 5, we conducted a generalised linear mixed-effects model with the logit link of the effect of task difficulty (target opacity from −5 to +5) and accuracy in the initial choice (coded as error: −0.5, correct: 0.5) on advice-taking behaviour (coded as reject: 0, accept: 1) on trials where the advice did not correspond to the participants’ initial choice, with counterpart participant (out of 20 participants) and observer number as random intercepts. To test whether advice-taking varied depending on whether the advice was given by another person as opposed to an artificial agent, we added to this model the agent as a fixed factor (coded as other person: −0.5, algorithm: 0.5).

For all models, in the case of convergence or singularity issues, we reduced the complexity of the random effects structure. Data distribution was assumed to be normal, but this was not formally tested. We followed up on significant effects with post-hoc comparisons using Bonferroni corrections.

### Reporting summary

Further information on research design is available in the Nature Portfolio Reporting Summary linked to this article.

## Results

All analyses reported here were pre-registered, unless noted otherwise. We first report the results of Experiment 1, and then each of the other experiments in turn; for a summary of main manipulations and results across all experiments, see Table 1.

**Table 1:** Summary of experimental designs and key findings

Exp	*N*	Task	H1: decision variables			H2: agent type
			Difficulty	Accuracy	RTs	
1	120	Visual perception	✔	✔	✔	✔ AI > human
			(interaction) ✔			
2	120	Visual perception	✔	✔	N/A	✔ AI > human
			✔			
3	60	Visual perception	✔	✔	N/A	✔ AI > human
			✔			
4	60	General knowledge	✔	✘	✔	✔ AI > human
			✔			
5	60	Visual perception	✔	✔	N/A	✔ AI > human
			✔			
6	60	Emotion perception	N/A	✔	✔	✘
7	60	Visual perception	✔	✔	N/A	✔ High > low
			✔			

The table lists each experiment (Exp), sample size (*N*), task domain, and primary effects on confidence attributions. Columns indicate whether confidence was significantly predicted by decision variables (H1: difficulty, accuracy, reaction time) and by agent type (H2: human vs. AI). ✔ = *p* < 0.05; ✘ = *p* > 0.05.

*RTs* Response times.

### Cues to others’ confidence

**Fig. 2:**
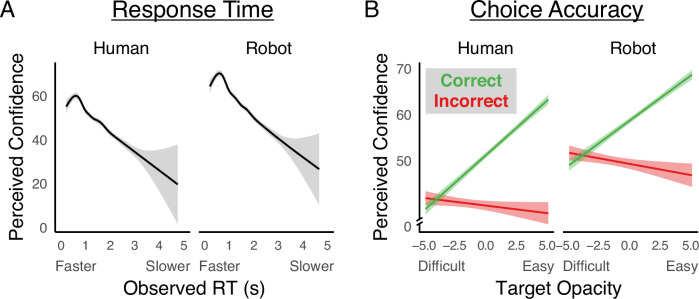
Evaluating others’ confidence. **A** Inferred confidence was affected by observed response time, with higher confidence inferences on trials where the agent responded faster. This pattern held both when observing other people (left) and robots (right). **B** Inferred confidence was generally higher for easier trials, but only when the agent was accurate (green) and not when inaccurate (red). As for response time, this pattern held both when observing other people (left) and robots (right). These data are from Experiment 1, but these patterns were robust across all experiments. Error bands represent 95% confidence intervals.

Attributions of confidence were sensitive to task parameters (i.e., task difficulty) as well as observed performance (i.e., accuracy and response times). As predicted, participants attributed higher confidence on easier trials where the target was presented at higher contrast (main effect of task difficulty: B = 0.54, SE = 0.14, *t*(19777) = 3.70, *p* < 0.001, CI = [0.25, 0.82]). Confidence estimates were also higher on trials where the agent responded faster (main effect of observed response time: B = −9.89, SE = 0.31, *t*(19729) = −32.31, *p* < 0.001, CI = [−10.49, −9.29]; Fig. 2A), and on trials where the agent responded accurately (mean = 55.31, SE = 1.16) vs. inaccurately (mean = 44.59, SE = 1.19; main effect of observed accuracy: B = 12.27, SE = 0.91, *t*(19788) = 13.51, *p* < 0.001, CI = [10.49, 14.05]). Interestingly, this effect was also sensitive to task difficulty: there was an interaction between observed accuracy and task difficulty (B = 2.61, SE = 0.29, *t*(19783) = 8.97, *p* < 0.001, CI = [2.04, 3.18]; Fig. 2B) wherein easier trials generated higher confidence estimates when the agent was correct (slope = 1.85, SE = 0.06, CI = [1.73, 1.97]), but not when the agent was incorrect (slope = −0.63, SE = 0.11, CI = [−0.84, −0.41]). This effect mirrors a classic statistical signature of confidence, the folded X pattern^39,42^, suggesting that people have a sophisticated understanding of the factors affecting others’ confidence.

We also investigated a possible relationship between self-directed and other-directed metacognition. We operationalised self-directed metacognition as metacognitive efficiency as participants were completing the detection task in Phase 1, and other-directed metacognition as the granularity of their attributions of confidence for other agents in Phase 2. Contrary to our hypothesis, there was no correlation between participants’ metacognitive efficiency and their capacity for other-directed metacognition (r(111) = −0.06, *p* = 0.556).

### Confidence in humans versus machines

**Fig. 3:**
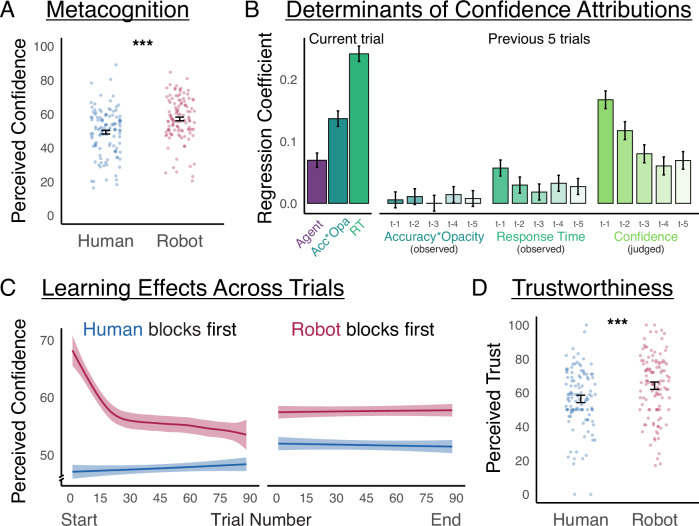
Attributions of confidence in humans and AI in Experiment 1. **A** Judgments of perceived confidence were higher for the artificial agent (red) compared to the human agent (blue). **B** In addition to agent (purple), these attributions were also predicted by behaviour observed on the current trial (left), as well as by confidence estimates on the previous trials (right). **C** The difference between agents was robust across experimental trials, and was especially strong at the beginning of the experiment for people who observed the other person first and the artificial agent later. **D** Perceived trustworthiness was also higher for robots compared to other people. Error bars correspond to mean ±95% confidence intervals, for (**A**) and (**D**) also subtracting out the shared variance. ***p < 0.001.

We next turned to our key question of whether and how attributions of confidence differed between humans and machines. Strikingly, perceived confidence was higher for robots (mean = 54.18, SE = 1.12) vs. other people (mean = 45.65, SE = 1.12), despite their performance being identical (main effect of agent: B = 11.82, SE = 0.88, *t*(19763) = 13.41, *p* < 0.001, CI = [10.09, 13.54]; Fig. 3A). This effect of agent also interacted with the main effects of difficulty (B = −0.82, SE = 0.28, *t*(19764) = −2.91, *p* = 0.004, CI = [−1.38, −0.27]) and response time (B = −2.30, SE = 0.56, *t*(19763) = −4.09, *p* < 0.001, CI = [−3.40, −1.20]), in that difficulty had a stronger effect on perceived confidence when the attribution was for another person (difficulty slope for other person = 0.78, SE = 0.09, CI = [0.61, 0.95], slope for robot = 0.43, SE = 0.09, CI = [0.26, 0.60]), while response time had a stronger effect on perceived confidence when the attribution was for the robot (response time slope for other person = −8.83, SE = 0.42, CI = [−9.65, −8.02], for robot = −11.13, SE = 0.41, CI = [−11.93, −10.33]). Overall, these analyses show that participants attributed greater confidence to the robot, despite the agents having identical performance—an ‘illusion of confidence’ in AI.

### Mechanisms of confidence attribution

To further deconstruct the relative contribution of observed behaviour and prior beliefs about the agents to confidence attributions, in an additional exploratory analysis we investigated how confidence attributions on a given trial were affected by observations on previous trials (Fig. 3B). We found that confidence attributions on a given trial were influenced by the type of agent and their behaviour on the current trial (main effect of agent, B = 3.73, SE = 0.32, *t*(18720) = 11.72, *p* < 0.001, CI = [3.11, 4.36]; main effect of accuracy, B = 10.61, SE = 0.38, *t*(18696) = 28.02, *p* < 0.001, CI = [9.87, 11.35]; main effect of difficulty, B = 0.60, SE = 0.06, *t*(18636) = 9.90, *p* < 0.001, CI = [0.48, 0.72]; main effect of response time, B = −10.31, SE = 0.27, *t*(18376) = −38.06, *p* < 0.001, CI = [−10.84, −9.78]; accuracy × difficulty interaction, B = 2.55, SE = 0.12, *t*(18649) = 21.20, *p* < 0.001, CI = [2.32, 2.79]; Fig. 3B, left), but also by their behaviour on previous trials. This was especially true for previous response times (trial −1: B = 2.44, SE = 0.28, *t*(18450) = 8.68, *p* < 0.001, CI = [1.89, 2.99]; trial −2: B = 1.26, SE = 0.28, *t*(18473) = 4.48, *p* < 0.001, CI = [0.71, 1.82]; trial −3: B = 0.78, SE = 0.28, *t*(18479) = 2.78, *p* = 0.006, CI = [0.23, 1.34]; trial −4: B = 1.39, SE = 0.28, *t*(18451) = 4.91, *p* < 0.001, CI = [0.83, 1.94]; trial −5: B = 1.16, SE = 0.28, *t*(18407) = 4.12, *p* < 0.001, CI = [0.61, 1.71]), and less so for the interaction between accuracy and difficulty (trial −1: B = −0.11, SE = 0.12, *t*(18656) = −0.87, *p* = 0.386, CI = [−0.34, 0.13]; trial −2: B = −0.20, SE = 0.12, *t*(18656) = −1.68, *p* = 0.094, CI = [−0.44, 0.03]; trial −3: B = 0.00, SE = 0.12, *t*(18655) = −0.04, *p* = 0.968, CI = [−0.24, 0.23]; trial −4: B = −0.27, SE = 0.12, *t*(18653) = −2.18, *p* = 0.029, CI = [−0.50, −0.03], and trial −5: B = −0.15, SE = 0.12, *t*(18655) = −1.21, *p* = 0.226, CI = [−0.39, 0.09]; Fig. 3B, middle). The strongest predictors of confidence attributions on any trial were confidence attributions on previous trials, which leaked into the current trial (trial −1: B = 0.17, SE = 0.01, *t*(18726) = 22.95, *p* < 0.001, CI = [0.15, 0.18]; trial −2: B = 0.12, SE = 0.01, *t*(18724) = 15.93, *p* < 0.001, CI = [0.10, 0.13]; trial −3: B = 0.08, SE = 0.01, *t*(18718) = 10.81, *p* < 0.001, CI = [0.07, 0.09]; trial −4: B = 0.06, SE = 0.01, *t*(18720) = 8.20, *p* < 0.001, CI = [0.05, 0.07]; trial −5: B = 0.07, SE = 0.01, *t*(18727) = 9.55, *p* < 0.001, CI = [0.05, 0.08]; Fig. 3B, right). This suggests that while attributions of confidence are sensitive to observed behaviour, they are also influenced by longer-timescale fluctuations, consistent with previously reported empirical patterns of confidence leak in self-directed metacognitive judgments^52,53^. This confidence leak phenomenon was also similar when interacting with both human and AI agents, as the impact of all variables from previous trials did not interact with agent type (agent × accuracy: trial −1: B = 0.22, SE = 0.76, *t*(18588) = 0.29, *p* = 0.768; trial −2: B = −0.59, SE = 0.76, *t*(18588) = −0.78, *p* = 0.438; trial −3: B = 0.41, SE = 0.76, *t*(18587) = 0.53, *p* = 0.594; trial −4: B = −0.16, SE = 0.76, *t*(18587) = −0.21, *p* = 0.835; trial −5: B = 0.18, SE = 0.76, *t*(18587) = 0.24, *p* = 0.810; agent × difficulty: trial −1: B = 0.06, SE = 0.12, *t*(18582) = 0.53, *p* = 0.594; trial −2: B = 0.16, SE = 0.12, *t*(18582) = 1.29, *p* = 0.198; trial −3: B = −0.04, SE = 0.12, *t*(18581) = −0.37, *p* = 0.713; trial −4: B = −0.08, SE = 0.12, *t*(18581) = −0.69, *p* = 0.490; trial −5: B = 0.15, SE = 0.12, *t*(18581) = 1.28, *p* = 0.199; agent × confidence: trial −1: B = 0.01, SE = 0.01, *t*(18601) = 0.58, *p* = 0.560; trial −2: B = 0.02, SE = 0.01, *t*(18595) = 1.67, *p* = 0.095; trial −3: B = −0.02, SE = 0.01, *t*(18595) = −1.10, *p* = 0.272; trial −4: B = −0.02, SE = 0.01, *t*(18597) = −1.09, *p* = 0.276; trial −5: B = 0.00, SE = 0.01, *t*(18604) = 0.35, *p* = 0.730; agent × response times: trial −1: B = 0.44, SE = 0.54, *t*(18578) = 0.82, *p* = 0.414; trial −2: B = −0.11, SE = 0.54, *t*(18578) = −0.20, *p* = 0.845; trial −3: B = 0.33, SE = 0.54, *t*(18578) = 0.61, *p* = 0.543; trial −4: B = −0.60, SE = 0.54, *t*(18577) = −1.10, *p* = 0.269; trial −5: B = −0.46, SE = 0.54, *t*(18577) = −0.85, *p* = 0.396), except for the accuracy × difficulty interaction on the third and fourth previous trials being stronger for the robot vs. the human, (trial −3: B = 0.51, SE = 0.24, *t*(18590) = 2.09, *p* = 0.037; trial −4: B = 0.46, SE = 0.24, *t*(18589) = 1.89, *p* = 0.058; and trial −1: B = 0.10, SE = 0.24, *t*(18589) = 0.42, *p* = 0.674; trial −2: B = −0.20, SE = 0.24, *t*(18589) = −0.84, *p* = 0.400, trial −5: B = −0.29, SE = 0.24, *t*(18589) = −1.20, *p* = 0.232). This suggests that attributions of confidence are sophisticated, in that they show integration over time similar to that previously observed for self-directed metacognition, but they are also biased, in that they are systematically higher for robots irrespective of both current and recently-observed behaviour.

In addition, we sought to further characterise this illusion of confidence in AI by exploring its time course across trials, as participants observe the agents make their choices (Fig. 3C). We found that in general, attributions of confidence became lower over the course of the blocks (main effect of trial number: B = −0.36, SE = 0.07, *t*(19769) = −5.14, *p* < 0.001, CI = [−0.50, −0.22]), with an illusion of confidence in AI being strongest at the beginning of the relevant experimental blocks (interaction between trial number and agent: B = −1.09, SE = 0.14, *t*(19769) = −7.80, *p* < 0.001, CI = [−1.36, −0.81]). This learning effect was especially strong for participants who observed another person before the robot (three-way interaction between trial number, agent, and block order: B = 1.40, SE = 0.22, *t*(19769) = 6.39, *p* < 0.001, CI = [0.97, 1.82]), suggesting that participants had an expectation that the robot would be more confident, especially immediately after watching the other person perform the task. Indeed, the effects of both trial number and agent were stronger for participants who watched the other person first (two-way interaction between trial number and block order: B = 0.34, SE = 0.11, *t*(19769) = 3.12, *p* = 0.002, CI = [0.13, 0.56]; between agent and block order: B = −11.60, SE = 1.48, *t*(19769) = −7.83, *p* < 0.001, CI = [−14.51, −8.70]). Overall, these results suggest that while participants were sensitive to observed behaviour, they also held a prior belief that machines would be more confident in their choices, especially after having observed humans first.

### From perceived confidence to social impressions

At the end of the experiment, we also asked participants to evaluate the agents’ trustworthiness and competence. As depicted in Fig. 3D, the artificial agent was rated as more trustworthy than the other person (*t*(112) = 3.51, *p* < 0.001, d = 0.33, CI = [0.14, 0.52]). The artificial agent was also rated as more competent, despite having identical performance to the human counterpart (*t*(112) = 7.46, *p* < 0.001, d = 0.70, CI = [0.49, 0.91]). These higher-level social impressions thus mirror the higher attributions of confidence—and indeed, an additional exploratory analysis revealed that attributions of confidence were positively correlated with both trustworthiness (r(224) = 0.23, *p* < 0.001) and competence (r(224) = 0.26, *p* < 0.001). This suggests that attributions of confidence are related to the formation of broader personality impressions, even for artificial systems.

### Experiment 2: Machine-like behaviour

The results of Experiment 1 show that people form estimates of other agents’ confidence guided by a sophisticated model of others’ behaviour, but that these attributions are consistently inflated for artificial agents. In Experiment 1, however, the behaviour of both agents was human-like, with accuracy and response times derived from the actual behaviour of “counterpart participants” who had previously taken part in the study. In Experiment 2, we sought to test the robustness of this illusion of confidence in AI by investigating whether it also arises when observing stereotyped, machine-like behaviour. This experiment was identical to Experiment 1, except that accuracy at each difficulty level was now based on the accuracy of a neural network trained to complete the perceptual discrimination task, and observed response times were now faster and less variable. As in Experiment 1, these generative functions were matched for both machine and human agents.

**Fig. 4:**
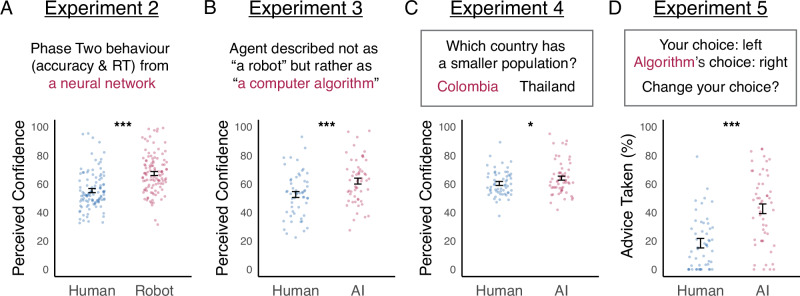
Establishing the generality of illusions of confidence in AI. For each experiment, we report key manipulations (top) and results (bottom). **A** Experiment 2: attributions of confidence to agents described as "a human" and "a robot". **B** Experiment 3: attributions of confidence to agents described as "a human" and "a computer algorithm". **C** Experiment 4: attributions of confidence in a general knowledge task. **D** Experiment 5: rates of advice-taking when the advisor was described as "a human" or "a computer algorithm". Perceived confidence was consistently higher for the artificial agent (red) compared to the human agent (blue) across all experiments. Error bars correspond to mean ±95% confidence intervals. *p < 0.05, ***p < 0.001.

As in Experiment 1, we found main effects of difficulty (B = 0.98, SE = 0.07, *t*(19074) = 13.28, *p* < 0.001, CI = [0.83, 1.12]) and accuracy (B = 8.97, SE = 0.45, *t*(19076) = 19.82, *p* < 0.001, CI = [8.08, 9.85]), as well as an interaction between these factors (B = 2.51, SE = 0.15, *t*(19076) = 17.04, *p* < 0.001, CI = [2.22, 2.80]) wherein easier trials generated higher confidence estimates when the agent was correct (slope = 2.23, SE = 0.07, CI = [2.10, 2.36]), but not when the agent was incorrect (slope = −0.28, SE = 0.13, CI = [−0.54, −0.02]). Most importantly, we again observed a main effect of agent (B = 12.07, SE = 0.44, *t*(19070) = 27.62, *p* < 0.001, CI = [11.21, 12.93]), with higher perceived confidence for the robot (mean = 64.15, SE = 1.08) vs. the other person (mean = 52.08, SE = 1.08; Fig. 4A). Agent type again interacted with difficulty (B = −0.68, SE = 0.14, *t*(19070) = −4.76, *p* < 0.001, CI = [−0.96, −0.40]), with a stronger relationship between difficulty and perceived confidence for the other person (slope = 1.32, SE = 0.10, CI = [1.13, 1.52]) compared to the robot (slope = 0.64, SE = 0.10, CI = [0.45, 0.84]).

We also replicated the (now pre-registered) effect of trial order, with stronger differences in the effect of agent at the beginning of each block, especially for participants who watched the other person before the robot (three-way interaction between trial number, agent, and block order: B = 1.64, SE = 0.22, *t*(19069) = 7.36, *p* < 0.001, CI = [1.20, 2.08]). Higher-level social impressions were again more positive for the robot, who was perceived as more trustworthy (*t*(108) = 4.26, *p* < 0.001, d = 0.41, CI = [0.21, 0.60]) and more competent (*t*(108) = 6.67, *p* < 0.001, d = 0.64, CI = [0.43, 0.84])—with these traits also being positively correlated with confidence attributions (trustworthiness r(216) = 0.32, *p* < 0.001; competence r(216) = 0.44, *p* < 0.001).

### Experiment 3: Machine-like descriptions

In both Experiments 1 and 2, the non-human agent was described and depicted as a robot (Fig. 1C). To ensure that attributions of confidence were not influenced by this specific presentation, we conducted a new experiment identical to Experiment 2, but where the agents were no longer explicitly pictured, and the non-human agent was described as a computer algorithm.

The results again replicated Experiments 1 and 2: we found main effects of difficulty (B = 1.17, SE = 0.11, *t*(9798) = 10.17, *p* < 0.001, CI = [0.94, 1.39]) and accuracy (B = 14.01, SE = 0.71, *t*(9799) = 19.75, *p* < 0.001, CI = [12.62, 15.40]), as well as an interaction between these factors (B = 3.67, SE = 0.23, *t*(9799) = 15.94, *p* < 0.001, CI = [3.22, 4.12]) wherein easier trials generated higher confidence estimates when the agent was correct (slope = 3.00, SE = 0.10, CI = [2.80, 3.21]), but not when the agent was incorrect (slope = −0.67, SE = 0.21, CI = [−1.07, −0.26]). Importantly, we again observed a main effect of agent (B = 8.79, SE = 0.70, *t*(9794) = 12.62, *p* < 0.001, CI = [7.43, 10.16]), with higher perceived confidence for the AI (mean = 56.83, SE = 1.83) vs. the other person (mean = 48.04, SE = 1.83; Fig. 4B). This is particularly striking given that in this design displays of agent behaviour were now identical, with the only difference being whether the agent was labelled as either a human or computer algorithm prior to the observation trials.

We also replicated the interaction between agent and difficulty (B = −0.86, SE = 0.23, *t*(9794) = −3.81, *p* < 0.001, CI = [−1.30, −0.42]), as well as the three-way interaction between trial number, agent, and block order (B = 1.00, SE = 0.36, *t*(9794) = 2.76, *p* = 0.006, CI = [0.29, 1.70]). As in Experiments 1–2, the artificial agent was seen as more trustworthy (*t*(55) = 5.13, *p* < 0.001, d = 0.69, CI = [0.39, 0.97]) and more competent (t(55) = 6.22, *p* < 0.001, d = 0.83, CI = [0.52, 1.13]) — with these traits also being positively correlated with confidence attributions (trustworthiness r(110) = 0.34, *p* < 0.001; competence r(110) = 0.33, *p* < 0.001).

### Experiment 4: General knowledge task

All experiments reported thus far involved a perceptual decision-making task, with participants deciding (and watching others decide) which of two stimuli contained a Gabor grating. However, past work has shown that attitudes towards algorithms can vary depending on the type of task at hand^12^. To establish that these effects are not specific to tasks involving perception, we investigated a different decision-making domain involving real-world knowledge^39^. Experiment 4 was thus similar to Experiment 3, except that the perceptual task was replaced by a general knowledge task where agents select which of two countries has a larger population (^54^; Fig. 4C).

The results were consistent with all previous experiments: we obtained main effects of difficulty (B = 1.53, SE = 0.53, *t*(10320) = 2.92, *p* = 0.004, CI = [0.50, 2.56]) and response time (B = −5.92, SE = 0.19, *t*(10324) = −30.69, *p* < 0.001, CI = [−6.29, −5.54]) on perceived confidence; the main effect of accuracy was not statistically significant (B = 1.94, SE = 1.29, *t*(10318) = 1.50, *p* = 0.135, CI = [−0.60, 4.47]), but we again found an interaction between difficulty and accuracy (B = 4.22, SE = 1.05, *t*(10319) = 4.03, *p* < 0.001, CI = [2.17, 6.27]) wherein easier trials generated higher confidence estimates when the agent was correct (slope = 2.71, SE = 0.32, CI = [2.09, 3.34]), but not when the agent was incorrect (slope = −1.43, SE = 0.52, CI = [−2.45, −0.41]). Importantly, we again observed a main effect of agent (B = 2.71, SE = 1.29, *t*(10313) = 2.10, *p* = 0.036, CI = [0.18, 5.24]), with higher perceived confidence for the AI agent (mean = 63.15, SE = 1.25) vs. the other person (mean = 59.12, SE = 1.25; Fig. 4C). We also replicated the three-way interaction between trial number, agent, and block order (B = 0.81, SE = 0.32, *t*(10319) = 2.51, *p* = 0.012, CI = [0.18, 1.45]). Finally, the artificial agent was again seen as more trustworthy (*t*(58) = 3.32, *p* = 0.002, d = 0.43, CI = [0.16, 0.70]) and more competent (*t*(58) = 4.88, *p* < 0.001, d = 0.63, CI = [0.35, 0.91])—with these traits also being positively correlated with confidence attributions (trustworthiness r(116) = 0.38, *p* < 0.001; competence r(116) = 0.43, *p* < 0.001). These results suggest that even in general knowledge tests, people expect algorithms to be more confident in their answers, despite the algorithm’s performance being equivalent to other agents. Taken together, our results demonstrate that the illusion of confidence in AI uncovered in previous experiments is not specific to perceptual decision-making but also arises in general-knowledge domains.

### Experiment 5: Influences on advice-taking

The experiments discussed so far show that people can form estimates of others’ confidence across different behavioural profiles, agent descriptions, and task domains. This was the case even though participants had no information regarding the sources of AI’s decisions, and even though errors about general knowledge were more easily verifiable than in the visual perception task. To further investigate these attributions of confidence, we designed a task where participants made perceptual choices, and were then given the opportunity to revise their choice after receiving ‘advice’ from one of two other agents^55^—either another person or a computer algorithm (Fig. 4D).

Advice-taking behaviour was consistent with the explicit ratings of perceived confidence from previous experiments. When receiving advice that was discordant with their initial choices (36.07% of all trials), participants sometimes reversed their choices (29.51% of discordant trials), and they did so more often when the task was more difficult (main effect of difficulty, B = 0.08, SE = 0.02, z = 5.08, *p* < 0.001, CI = [0.05, 0.11]) and when they were initially wrong (main effect of initial accuracy, B = 0.99, SE = 0.10, z = 9.79, *p* < 0.001, CI = [0.79, 1.19]). There was also an interaction between difficulty and initial accuracy (B = 0.20, SE = 0.03, z = 6.23, *p* < 0.001, CI = [0.14, 0.26]) wherein initial choices were revised more often on more difficult trials when the initial choice was incorrect (slope = 0.18, SE = 0.02, CI = [0.14, 0.23]), but not when correct (slope = −0.02, SE = 0.02, CI = [−0.06, 0.03]). Importantly, we observed a main effect of agent (B = 1.37, SE = 0.10, z = 13.08, *p* < 0.001, CI = [1.16, 1.58]), with a greater number of choice revisions after receiving advice from the AI (mean = 1.87, SE = 0.20) vs. the other person (mean = 0.45, SE = 0.19; Fig. 4D). We also replicated the three-way interaction between trial number, agent, and block order (B = 0.12, SE = 0.05, z = 2.37, *p* = 0.018, CI = [0.02, 0.22]). Finally, we found similar effects of agent on higher-level social impressions — with the artificial agent being perceived as more trustworthy (*t*(51) = 4.05, *p* < 0.001, d = 0.56, CI = [0.27, 0.85]) and more competent (*t*(51) = 3.60, *p* < 0.001, d = 0.50, CI = [0.21, 0.79]), in line with the profile of advice taking (trustworthiness r(102) = 0.50, *p* < 0.001; competence r(102) = 0.48, *p* < 0.001). This experiment thus showed that participants do not just attribute higher confidence to artificial agents, but are also more willing to defer to their advice.

### Experiment 6: A subjective task

While the illusion of confidence was robust across tasks involving perceptual decision-making (Experiments 1–3) and general knowledge (Experiment 4), both of these tasks involve decisions that can be objectively correct or incorrect. To further probe the boundary conditions of this effect, we ran a new experiment involving a more subjective task, namely emotion categorisation. On each trial, participants saw a face displaying one of six possible emotions: fear, anger, disgust, happiness, sadness, or surprise. They then observed another agent choose which emotion the face seemed to display, and indicated how confident the agent seemed in that choice. This paradigm was thus similar to Experiments 1–5, but probed inferences of confidence in subjective decisions.

**Fig. 5:**
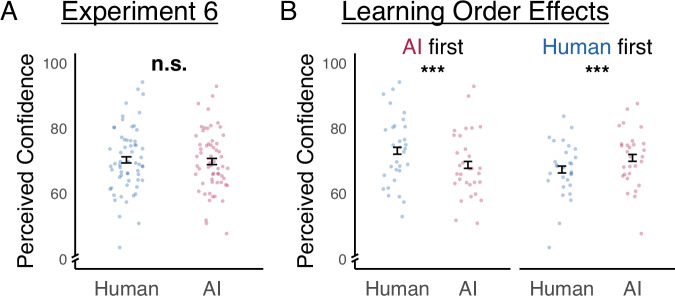
Attributions of confidence in a subjective task. **A** Inferences of confidence did not differ significantly between humans (blue) and artificial agents (red) overall. **B** These attributions, however, differed depending on task order, such that they were greater for humans compared to artificial agents for participants who observed the artificial agent first. Error bars correspond to mean ±95% confidence intervals. ***p < 0.001, n.s. not significant.

Attributions of confidence were again sensitive to observed performance and task characteristics. There was a main effect of accuracy (B = 16.86, SE = 0.93, *t*(10744) = 18.06, *p* < 0.001, CI = [15.03, 18.69]) and response time (B = −4.53, SE = 0.09, *t*(10731) = −52.06, *p* < 0.001, CI = [−4.70, −4.36]). There was also a trending interaction between these factors (B = −0.31, SE = 0.17, *t*(10741) = −1.89, *p* = 0.059, CI = [−0.64, 0.01]), wherein faster trials generated higher confidence estimates more strongly when the agent was correct (slope = −4.69, SE = 0.10, CI = [−4.89, −4.49]), compared to when the agent was incorrect (slope = −4.38, SE = 0.14, CI = [−4.65, −4.11]). Overall, and unlike in Experiments 1–5, inferences of confidence did not differ significantly between humans and artificial agents (main effect of agent: B = −0.27, SE = 0.92, *t*(10736) = −0.29, *p* = 0.771, CI = [−2.06, 1.53]; Fig. 5A). There were also no significant differences between agents in final assessments of trustworthiness (*t*(59) = 1.13, *p* = 0.264, d = 0.15, CI = [−0.11, 0.40]) or competence (*t*(59) = −0.81, *p* = 0.419, d = −0.10, CI = [−0.36, 0.15])—although these impressions were again positively correlated with confidence attributions (trustworthiness r(118) = 0.21, *p* = 0.023; competence r(118) = 0.32, *p* < 0.001).

The analyses of block order and trial number, however, revealed a more nuanced effect of agent, which varied throughout the experiment (Fig. 5B). First, inferences of confidence increased as participants observed choices over the course of each block (main effect of trial number: B = 0.54, SE = 0.12, *t*(10734) = 4.34, *p* < 0.001, CI = [0.30, 0.78]), and this increase was greater for the artificial agent (slope = 0.56, SE = 0.13, CI = [0.31, 0.81]) than for the human (slope = 0.37, SE = 0.13, CI = [0.12, 0.62]; interaction between agent and trial number: B = 0.49, SE = 0.25, *t*(10734) = 1.98, *p* = 0.048, CI = [0.00, 0.98]). The addition of block order in the model now also revealed a main effect of agent (B = −7.03, SE = 1.54, *t*(10734) = −4.57, *p* < 0.001, CI = [−10.05, −4.01]), with higher confidence attributed to humans (mean = 70.3, SE = 1.18) compared to artificial agents (mean = 69.9, SE = 1.18). But critically, this main effect interacted with block order (B = 11.23, SE = 2.21, *t*(10734) = 5.07, *p* < 0.001, CI = [6.89, 15.57]): attributions of confidence were greater for humans compared to artificial agents for participants who observed the artificial agent first (mean human = 73.19, SE = 1.64; artificial agent = 68.86, SE = 1.64; z = 6.08, *p* < 0.001), but this effect reversed for those who watched the human first (mean human = 67.42, SE = 1.69; artificial agent = 71.00, SE = 1.69; z = −4.86, *p* < 0.001).

This experiment thus revealed that attributions of confidence in a subjective task are similar across humans and artificial agents, contrary to the pattern observed for perceptual and general-knowledge tasks investigated in Experiments 1–5.

### Experiment 7: A causal mechanism

**Fig. 6:**
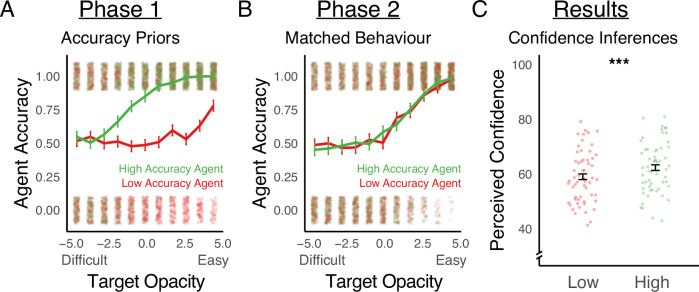
A mechanism underlying illusions of confidence in AI. **A** Participants observed two agents that differed in accuracy (Phase 1) and (**B**) later reported their impressions of the agents’ confidence in trials with matched behaviour (Phase 2). **C** Perceived confidence was higher for the agent associated with a ‘high accuracy prior’ (green) compared to a ‘low accuracy prior’ (red). Error bars correspond to mean ±95% confidence intervals. ***p < 0.001.

Our previous experiments revealed a powerful illusion of confidence in objective tasks (Experiments 1–5), but not in more subjective ones (Experiment 6). The fact that attributions of confidence were modulated by task domain led us to hypothesise that this illusion may be rooted in prior beliefs about artificial agents’ abilities—such that it is greatest for tasks typically associated with artificial agents (e.g., image detection), but not for those that seem more subjective (e.g., emotion categorisation). That is, people may believe that artificial agents are more skilled than humans at visual perception, but not emotion discrimination, and this prior belief may in turn influence confidence attributions (see also ref. ^56^). To test this hypothesis, we measured attributions of confidence to agents that observers believed were more or less skilled. These priors were induced via an initial phase of the experiment (Phase 1, ‘Observation’) where participants watched artificial agents perform at different mean accuracy levels (Fig. 6A). In a second phase (Phase 2, ‘Inferences’), participants observed the two agents, now with matched behaviour, and reported their inferences of the agents’ confidence (Fig. 6B, C).

As in previous experiments, inferences of confidence were sensitive to observed behaviour: there was a main effect of accuracy (B = 13.70, SE = 0.79, *t*(4963) = 17.35, *p* < 0.001, CI = [12.15, 15.24]), a main effect of difficulty (B = 0.74, SE = 0.13, *t*(4958) = 5.70, *p* < 0.001, CI = [0.49, 1.00]), and an interaction between these factors (B = 2.90, SE = 0.26, *t*(4963) = 11.10, *p* < 0.001, CI = [2.39, 3.42]) wherein easier trials generated higher confidence estimates when the agent was correct (slope=2.20, SE = 0.13, CI = [1.95, 2.45]), but not when the agent was incorrect (slope = −0.71, SE = 0.23, CI = [−1.15, −0.26]). Importantly, there was a main effect of prior accuracy (B = 3.75, SE = 0.79, *t*(4954) = 4.78, *p* < 0.001, CI = [2.21, 5.29]), such that inferences of confidence in Phase 2 were greater for the agent who showed higher accuracy in Phase 1 (mean = 58.88, SE = 1.22) compared to the agent who showed lower accuracy (mean = 55.13, SE = 1.21).

Further analyses of trial order revealed a trending interaction between agent prior accuracy and trial number in Phase 2 (B = −0.54, SE = 0.28, *t*(4953) = −1.92, *p* = 0.055, CI = [−1.09, 0.01]), with a stronger decrease in confidence inferences for the high accuracy prior agent (slope = −0.73, SE = 0.16) compared to the low accuracy prior agent (slope = −0.29, SE = 0.16). Finally, the high accuracy prior agent was seen as more trustworthy (*t*(56) = 4.06, *p* < 0.001, d = 0.54, CI = [0.26, 0.81]) and more competent (*t*(56) = 5.08, *p* < 0.001, d = 0.67, CI = [0.38, 0.96])—with these traits also being positively correlated with confidence attributions (trustworthiness r(112) = 0.33, *p* < 0.001; competence r(112) = 0.27, *p* = 0.004).

Our experimental manipulation of priors over agent performance thus resulted in different inferences of confidence, even within the same subjects, such that participants attributed greater confidence to algorithms they believed to be more accurate. Inferences of confidence are thus at least in part driven by prior beliefs about the accuracy of other agents, furnishing a causal psychological mechanism explaining illusions of confidence in artificial systems.

## Discussion

Successful cooperation requires an understanding of others’ mental states such as their feelings, goals, and beliefs, with previous work in psychology and neuroscience exploring this rich capacity for mental state attributions. Here we consider that in addition to others’ *cognitive* states, social interactions also require that we perceive others’ *metacognitive* states, such as how confident others are in their beliefs and decisions. Across seven experiments, we found that when watching other agents make decisions, people form sophisticated estimates of their confidence, influenced by both task variables (e.g., difficulty) and observed behaviours (e.g., response time and accuracy on each trial). Strikingly, however, people also consistently ascribed higher confidence to the decisions of artificial agents, compared to those of performance-matched humans. This illusion of confidence emerged in perceptual decisions from both human- and machine-like behaviour (Experiment 2), and from both anthropomorphic and non-anthropomorphic agent descriptions (Experiment 3). When tested in other decision-making domains, these effects were consistent for objective choices (general knowledge; Experiment 4), but not for more subjective ones (emotion categorisation; Experiment 6). Indeed, an experimental manipulation revealed that attributions of confidence are rooted in prior beliefs about accuracy, revealing a causal mechanism underlying illusions of confidence in AI (Experiment 7).

The illusion of confidence uncovered in these experiments was especially striking given that in each experiment, the behaviour of the artificial agents was in fact equated to the human agents: the observed accuracy and response times were drawn from the same generative functions, such that any difference in perceived confidence solely reflected participants’ assumptions about the agents. Indeed, further analyses of learning effects revealed that this bias was stronger at the beginning of the experimental blocks, and diminished thereafter as participants observed the agents’ behaviour. This updating may in part reflect the feedback on the accuracy of their confidence attributions that was provided to participants every few trials, which participants may have used to guide their estimates. However, we consider this unlikely, as the feedback was provided as a summary statistic (i.e., a cumulative total) and was non-directional (i.e., both overestimation and underestimation of confidence would yield the same feedback outcome). In addition, despite a reduction in its magnitude, the illusion of confidence remained robust toward the end of the experiment, suggesting that it was relatively resistant to feedback. Instead, we suggest that this reduction reflects a discrepancy between participants’ prior beliefs about an agent (e.g., that the algorithm would excel in general knowledge) and its behaviour (which was sometimes inaccurate). This was further confirmed by an experimental manipulation of prior beliefs about performance in Experiment 7, which demonstrated that identical decisions were perceived as more confident when participants held a prior belief that the algorithm was more accurate.

The role of prior beliefs about performance is especially intriguing as it suggests that beliefs about other systems’ abilities can shape not just whether an advisor is generally regarded as trustworthy and whether individual answers are deemed likely to be true, but also the mental states attributed to the agents. Higher attributions of confidence may, in turn, reinforce the perceived accuracy of the responses, creating a feedback loop that amplifies both trust in the system and in its suggestions^57^. This notion was supported by analyses of task order in Experiment 6, where inferences of confidence were greater for artificial agents when these were observed after humans. In post-experiment debriefing, participants reported they had been surprised by some of the choices they observed from the other humans, noting how some of these selections seemed counterintuitive. The fact that observing human errors increases attributions of confidence to artificial agents suggests that differences in attributed confidence may reflect prior beliefs about the agents’ *relative* accuracy, such that they are initially greater for agents presumed to be more accurate in this subjective task (i.e., humans), but increase for artificial agents after observing human errors. It also remains possible that, in addition to overall beliefs about accuracy, differential attributions of confidence to humans and AI systems may have resulted from observers’ beliefs about their different decision processes (e.g., AI’s algorithmic or logical versus humans’ intuitive and probabilistic reasoning). While we suspect that, ultimately, assumptions about decision processes may impact attributed confidence to the extent that they impact accuracy (e.g., an answer from logic being more likely to be accurate than one from intuition), future work may examine the relative contribution of these factors by varying properties of the internal functionality of the system independent of its performance output^58^.

The impact of prior beliefs about accuracy demonstrated in the causal manipulation in Experiment 7 may also explain why illusions of confidence were strongest in tasks where AI is presumably highly accurate (e.g., visual detection in Experiments 1–3), and were minimised in more subjective tasks (e.g., emotion categorisation in Experiment 6). In particular, participants may have a prior belief that AI systems may be less accurate at emotion recognition given the common intuition that machines, unlike humans, do not themselves have the capacity to experience emotion^59^. We note, however, that a prior belief that humans may be more accurate in this task did not result in a reversal of the illusion, as observers did not perceive humans to be more confident than AI. This suggests that both performance priors and a general bias toward perceiving AI as more confident likely work together to produce the observed effect. Alternatively, the more subjective nature of the task may have led observers to rely on immediate behavioural cues (e.g., RTs) that were identical across agent types, rather than a priori beliefs about agents’ accuracy, resulting in no statistically significant differences between agent types. Confidence about subjective quantities may also rely more on assessments of choice consistency than objective correctness^60,61^. Finally, the lack of a reliable difference between human and AI confidence in this domain may also reflect properties of the task itself; for example, participants may be attributing overall lower confidence to all agents given the more ambiguous nature of the task, resulting in a floor effect. This possibility seems unlikely, given that mean confidence ratings were comparable across experiments, and if anything, were *higher* in emotion categorisation (average Exp. 1 = 53.22; Exp. 2 = 61.30; Exp. 3 = 57.29; Exp. 4 = 62.20; Exp. 6 = 70.15). Rather than changes in overall confidence, we thus interpret these findings as reflecting either different ability priors, such that AI systems were no longer believed to be more accurate, or a shift in cue weighting, where observers ceased to rely on prior beliefs and rather based confidence attributions on directly observable behavioural indicators (e.g., reaction time and choice consistency) that did not differ across agents.

In addition to prior beliefs, attributions of confidence also reflected trial-by-trial variations in performance in each experiment (Table 1), revealing a rich capacity for metacognitive attributions. In particular, participants were able to intuitively perceive other agents’ confidence based on observed variables that past research on metacognition has shown to affect (self-)confidence. For example, in Experiments 1 and 4, participants used response time as a cue to confidence, with faster response times leading to higher confidence attributions. This result is consistent with past work showing that response times can function as cues not only to confidence^62^, but also to mental states including cognitive effort^63^, abstract preferences^20,64^, personality traits^65^, and moral character^66^. But rather than being parametrically dependent on just one variable, attributions of confidence incorporated several cues as well as their interactions. For example, in all experiments, participants used task difficulty as a cue to confidence, with higher confidence attributions for easier trials—but only when the decisions were correct, mirroring a classic statistical signature of confidence^39,42^. Importantly, comparisons with participants’ own metacognitive profiles in Experiment 1 confirmed that these attributions did not just reflect participants’ own confidence but instead reflected novel inferences about others’ behaviour. This form of ‘folk metacognition’—how people attribute metacognitive states such as confidence to other agents—adds a new dimension to a growing body of evidence for how we achieve a naïve understanding of higher cognitive processes such as awareness and attention^67–69^.

Of note, in all experiments participants reported that the computer algorithm was not just more confident in each choice, but also overall more trustworthy and more competent compared to the matched human counterpart. While future work is needed to probe possible causal relationships between perceived confidence and social impressions, this result suggests that illusions of confidence may play an important role in regulating trust in interactions. For example, in tasks where human operators can choose to delegate tasks to automated agents such as clinical decision support systems or autonomous vehicles^70^, users may choose to delegate more often if higher confidence is attributed to a potential collaborator. The results of Experiment 5 are especially relevant in this respect, as they suggest that attributions of confidence affect advice-taking in a scenario akin to AI support and assistance. And beyond trust in other systems, illusions of confidence may also impact participants’ own metacognitive processes. For example, past work has shown that when making decisions in groups, individuals adapt their own confidence estimates to align with others’, allowing for appropriate weighting of confidence in the presence of bias^18^. While alignment with humans is often based on reliable cues embedded in communication (e.g., speech prosody^24^), such cues are typically absent in AI output, and alignment with machines may thus rely more heavily on prior beliefs rather than momentary cues. As a result, a tendency to over-attribute confidence to AI systems may lead to a disruption of metacognitive processes in users.

Given the role of confidence in regulating interpersonal trust and decision-making, our findings of an illusion of confidence in AI systems highlight the necessity for developing solutions for artificial metacognition that allow AI agents to appropriately communicate (potentially low) degrees of confidence in their behaviour^71^. Future work could explore types of confidence broadcasts (e.g., explicit numerical estimates, or implicit visual indicators) to facilitate alignment in human-AI interactions, and how these might help counter inaccurate priors^38^. Beyond highlighting the importance of integrating confidence estimates in AI systems, the studies reported here reveal specific determinants of confidence attributions, including prior beliefs and decision speed, which are likely to mislead observers and could be targeted in AI design to prevent erroneous attributions. For instance, the results of accuracy priors in Experiment 7 suggest that inflated attributions of confidence can be prevented by communicating the overall accuracy of the system. Similarly, the systems could automatically detect cases where users are likely to infer high confidence (e.g., on fast deliberation times), to flag potential divergences between objective accuracy and users’ perceptions of performance.

The types of confidence attributions explored in the current study may also have rich connections to our robust capacity for self-directed metacognition^72^. This link was partially explored in Experiment 1: we expected that participants with better metacognitive insight into their performance would also form more fine-grained confidence attributions about others, but did not find any significant relationship between these measures. This result is relevant to past work on action understanding, which posits that inferences of others’ mental states from their actions are based on observers’ own motor system. For example, past work has shown that inferences of others’ confidence are relative to participants’ own kinematic profiles, likely because these judgments are based on simulations of participants’ own motor system^23^. The current experiments differed from these previous paradigms as participants did not have access to movement kinematics. Nevertheless, our findings demonstrate that judging metacognitive states in others may not require access to participants’ own metacognitive system, and can in principle be based on behavioural observations alone. Future work could probe possible associations and dissociations between self- and other-directed metacognition, and the relative weight given to external cues such as response times^21^ and contextual factors, as opposed to internal models of self and other (ref. ^41^; see also ref. ^17^). An especially intriguing future direction in this domain is the study of child development, given proposals that metacognition may be scaffolded by the development of social cognition in childhood^73,74^.

### Limitations

Along with these implications, we note several limitations of the current studies that should be investigated in future research. First, the studies reported here tested illusions of confidence across different agent descriptions, from robots (Experiments 1–2) to computer algorithms (Experiments 3–7), but AI systems can vary widely in terms of their functions and appearance. Future work may further examine how participants interpret these labels and potential assumptions they might have about different types of artificial agents, particularly with respect to their capacities and abilities, which in turn would influence attributions of confidence. Second, in all current studies the agents’ accuracy in the inference task was matched to that of the observers. While this design choice allowed us to isolate attributions of confidence from objective performance differences, future work should examine conditions in which agents display higher or lower ability than the observer. The present results already highlight a critical role of perceived agent accuracy, both at the trial level (as reflected in the main effects of accuracy that emerged in all experiments), and at the global level (as demonstrated by the causal manipulation of accuracy in Experiment 7). We further speculate that these effects may interact with observers’ own ability, such that more skilled observers might attribute lower confidence to others because they are better able to detect inaccuracies, whereas less skilled observers might over-attribute confidence to others when they cannot reliably discriminate errors.

We also note that the current tasks involved minimal social interactions and other agents were described in relatively anonymous and generic terms (e.g., “another participant who previously completed this task”). However, everyday life typically involves repeated interactions with known others who may differ in baseline metacognitive abilities, above and beyond the current context. For example, past work has shown robust individual differences in metacognition^75^ related to variation in mental health^76^ and personality traits such as dogmatism and radicalism^77,78^, raising the possibility that perceived confidence might depend not just on temporary states (e.g., how fast someone responds in that instance), but also on knowledge of more general personality traits (e.g., how fast someone responds in that instance compared to how they typically respond in the face of uncertainty).

## Conclusions

In sum, these results demonstrate that people can form robust impressions of the metacognitive states of other agents. Beyond demonstrating the effect of candidate cues that affect attributions of confidence (e.g., response times), we also highlight a striking illusion of greater confidence when attributing confidence to AI, even when objective performance is matched to humans. We expect this confidence illusion to have significant consequences for human-AI interaction, possibly leading to unwarranted trust and reliance on AI advice. In turn, countering this bias will require the development of accurate systems for artificial metacognition, and reliable means of communicating metacognitive states to humans. Collectively, our results point to a rich capacity for metacognitive inference from others’ behaviour, and highlight the importance of ‘folk metacognition’ in social interactions.

## Supplementary information

Transparent Peer Review file

Reporting Summary

## Data Availability

Anonymised raw data for all experiments are openly available on the Open Science Framework (OSF) website at this link: https://osf.io/8tphs/.

## Code availability

Analysis code for all experiments is openly available on the OSF website at this link: https://osf.io/8tphs/.
