# Supplementary material for: Beliefs about accuracy shape confidence attributions to humans and artificial agents
Source: Commun Psychol. 2026 Apr 22;4:114. doi: 10.1038/s44271-026-00445-4 (PMC13461344; doi:10.1038/s44271-026-00445-4)
Supplement: Supplementary file 1 [file 44271_2026_445_MOESM1_ESM.pdf]

nature portfolio

Peer Review File

Attributing Confidence to Humans and Artificial Agents: Beliefs About Accuracy Shape Metacognitive Inference

Corresponding Author: Dr Clara Colombatto

This file contains all editorial decision letters in order by version, followed by all author rebuttals in order by version.

Version 0:

Decision Letter:

** Please ensure you delete the link to your author homepage in this e-mail if you wish to forward it to your coauthors **

Dear Dr Colombatto,

Thank you for your patience during the peer-review process. Your manuscript titled "Illusions of Confidence in Artificial Systems" has now been seen by 4 reviewers, and I include their comments at the end of this message.

They find your work of interest but raised some important points. We are interested in the possibility of publishing your study in Communications Psychology, but would like to consider your responses to these concerns and assess a revised manuscript before we make a final decision on publication.

We therefore invite you to revise and resubmit your manuscript, along with a point-by-point response to the reviewers. Please highlight all changes in the manuscript text file.

The reviewers make a number of presentational suggestions. You have different options on how to address these, but editorially, we encourage authors to include Figures that give readers a genuine sense of the data, and we ask for all main analyses to be included in the main paper.

I am attaching an Editorial Requests Table that details critical reporting requirements for the revised manuscript. Please attend to each item and ensure your manuscript is fully compliant. If your revised manuscript is not aligned with these requests on major issues, such as those concerning statistics, it may be returned to you for further revisions without re-review. Aligning your manuscripts with our requirements will significantly speed up future steps.

Please submit the following items:

- Revised manuscript
- Point-by-point response to the referees' comments
- Cover letter (as a separate document)
- Nature Research Reporting Summary
- Completed Editorial Request Table (attached).

via this link: Link Redacted .

** This url links to your confidential home page and associated information about manuscripts you may have submitted or be reviewing for us. If you wish to forward this email to co-authors, please delete the link to your homepage first **

Additional guidance is available in our style and formatting guide Communications Psychology formatting guide.

We hope to receive your revised paper within 8 weeks; please let us know if you aren't able to submit it within this time so that we can discuss how best to proceed. If we don't hear from you, and the revision process takes significantly longer, we may close your file. In this event, we will still be happy to reconsider your paper at a later date, provided it still presents a significant contribution to the literature at that stage.

We would appreciate it if you could keep us informed about an estimated timescale for resubmission, to facilitate our planning.

We look forward to seeing the revised manuscript and thank you for the opportunity to review your work.

Best regards,

Marike

Marike Schiffer, PhD
Chief Editor
Communications Psychology

REVIEWER EXPERTISE:
The referees have expertise in decision making, metacognition, and human-AI interaction

REVIEWER REPORTS:

Reviewer #1 (Remarks to the Author):

This paper investigates how humans infer confidence from other agents, and specifically how this process operates differently when the agent is artificial (AI) rather than human. Across seven pre-registered experiments, the authors demonstrate a consistent and systematic "illusion of confidence" in AI: human participants tend to attribute higher confidence to artificial agents compared to human ones, even when the two exhibit identical behavior.

I think this is an interesting and useful set of experiments. The experiments span a range of tasks including perceptual decision-making, general knowledge quizzes, emotion categorization, and advice-taking scenarios. This variety of tasks makes the findings robust and more interesting. Participants over-attribute confidence to AI across multiple contexts, and the effect is mediated by prior beliefs about AI competence. The authors propose that these results point to a fundamental cognitive bias that could have broad implications for human-AI collaboration and trust in automated systems.

I commend the authors for their pre-registration. The experiments are sufficiently powered, and have transparent exclusion criteria and statistical analyses. The manuscript is generally well written.

I have a few main concerns/recommendations. First, I think the theoretical framework is lacking. The set of experiments are incremental and appear quite exploratory with little or no common framework regarding the factors influencing confidence judgments. I find the connection to "metacognition" unclear or confusing. The "folk metacognition" as a way in which laypeople perceive and interpret confidence in others, including AI, is not well-founded on the factors investigated in the various experiments.

Also, related, the absence of an illusion in emotion categorization in Experiment 6 is intriguing, but the explanation could benefit from further elaboration. For instance, could participants simply perceive emotion judgments as more ambiguous, reducing confidence judgments overall?

Second, just like the authors present some common figures across experiments, a summary table reporting the main results and statistics across experiments would be useful. It is very easy to get lost in reading with the multiple numbers and tests in-between.

Finally, I recommend that the authors expand on the implications of these findings for AI design. How do these findings translate into real-world systems? I would like to see a more concrete proposal for mitigating the illusion of confidence.

Reviewer #2 (Remarks to the Author):

I enjoyed reading this paper on confidence attributions. Naïve participants observed the behaviour of (assumed, as per experimenter instructions) either human or artificial agents, the latter corresponding to robots, neural networks, or algorithms. The observed behaviour concerned a perceptual task (matching noise patterns to a Gabor patch) or a general knowledge task (questions concerning country population) or an emotional categorisation task. Behaviour was matched between human and artificial agents. Note, in most experiments the target DV was judgments of how confident the agent (human or artificial) would be in their judgments; in one case, there was a DV of whether the participant would take advice from an agent (the participant would make a judgment and then given the opportunity to revise the judgment, based on agent feedback). There were several checks that the experiments worked sensibly, for example, confidence attributions went hand in hand with lower RT and higher accuracy. The main research question of the researchers is whether confidence attributions would vary between (assumed) human and artificial agents. The main finding is that, fairly uniformly, artificial agents would be considered as having more confidence in their responses, compared to human agents. There was one exception, namely there was no difference in confidence attributions, when the task was one of emotional categorisation.

There are many positives in this work. The overall result is interesting. The methodology is very meticulous and the range of manipulations extensive and convincing. I think this work will interest (and excite) many readers. Given the careful and rigorous methodology, I think most readers will accept the presented conclusions. I do have some objections – these do not attest to the quality of the research, rather to the way I interpreted the results. I do not intend these objections to be seen as undermining publication prospects; I rather offer them as indications of the kind of debate that this paper is likely to generate.

Foremostly, the authors label their result as an illusion of confidence – that is, an illusion that an AI agent would be more confident in their decisions than a corresponding human agent, even if observed behaviour between the two kinds of agents is identical. As an aside, did participants notice / comment on the similarities between ostensibly human and artificial agents? In any case, I could not agree with the label ‘illusions’. I think confidence in a decision / judgment is a function of many factors: one of the factors is content, so that for very simple decisions, one can imagine that the generating agent would be confident. However, another key factor, and one that does not seem to be acknowledged in this work, is the process via which an answer is generated. For example, if we consider a long division, e.g., 5345633 divided by 3452, both a human and an artificial agent (at the simplest level, a calculator!) may arrive to the same answer. However, would we not have more faith in the artificial agent, on the back of assumptions that artificial agents are carrying out this process on the basis of multiply verified and logically correct algorithmic processes? Likewise, say, in considering an ailing patient, both a naïve observer and an expert doctor might offer the same advice, but would it not be the case that we would attribute more confidence to the doctor?

Another related issue is that equating performance between human and artificial agents would very much be a function of what kind artificial agent we are talking about. As a trivial example, a calculator is extremely capable in arithmetic but useless in language tasks. Therefore, the importance of equating behaviour between artificial and human agents is suspect in this way too; the only way I could see of addressing this issue is through more detail of the kind of artificial agent relevant in each experiment (more so than is currently provided, noting that, for example, for an average naïve participant algorithm or neural networks may not mean much).

Another issue is that the main DV – attributions of how confident an agent is – struck me as a little bit odd, in that it assumes that both human and artificial agents share a similar mechanism of self-reflection and self-evaluation. In some cases, this is clearly less meaningful: for example, we could not ask whether a calculator is confident in the response it provides for long division. Some care is needed here, because there is a danger that, in some respects, the research question could be criticised as suspect. Note, I am curious as to why the authors did not consider instead the question of trust in human vs AI agents. This is done in one of the experiments, where participants were asked to decide whether to revise their response, but it is certainly not one of the main questions. I do appreciate that what I am interested in is not necessarily what the authors are interested in; however, as per the above, I do think that there is a need to explain what it means (what the authors think it means) for an artificial agent to be confident or not in a decision (and so what attributions participants are making or think they are making).

Minor methods issues: the analyses on changes with time are interesting, but I was unsure what to make of them. It is well-known that there are question order effects or evaluation biases (that is, biases from considering some earlier information and responding to it). It is possible that some readers might feel that the analyses are a little superfluous. Perhaps either explain better why you are doing them or move them to supplementary material? Also, with the mixed effects analyses, it would be helpful to explain more clearly whether you employed slopes and if yes for which effects; and, if you did employ slopes, what kind of covariance matrix you assumed.

Reviewer #3 (Remarks to the Author):

The paper presents seven pre-registered studies investigating human attributions of confidence to artificially intelligent systems (AI) in comparison to performance-matched human counterparts. The authors’ primary finding is that people ascribed higher confidence to the decisions of AI agents than humans. This effect is demonstrated across agent descriptions and various objective perceptual tasks, is reduced under a subjective emotional identification task, and is shown to be influenced by prior beliefs about AI capabilities.
We found this manuscript to be a comprehensive and well-executed study that addresses an important, relevant, and timely area of research in human-AI interaction. Overall, the manuscript is clear and well-written. It presents a robust set of experiments that contribute meaningfully to our understanding of human perception of AI’s metacognitive states. We have a

few comments on the interpretation of specific experimental outcomes and a suggestion on the data used in a figure.

Major suggestions:
1. Generalizability beyond matched performance: The study's design matches the performance of the human and AI counterparts to the performance of the observer/participant. This is a nicely designed control to examine the phenomena across these several studies. However, it raises a question about the generalizability or the extent of the illusion of confidence when humans interact with human and AI counterparts whose performance levels are notably different from their own (better OR worse). Many of the nuanced findings in the literature around human-AI trust are often moderated by such nuanced relationships. We suggest that the discussion section considers the implications of this design choice for the broader applicability of this finding.

2. Following from the previous point - Figure 2 demonstrates the nice effects of changes to perceived confidence (A) for RT and (B) task difficulty by correct and incorrect responses and captures an intuitive finding for the relationship between confidence attributions and RT/accuracy. Given that the authors report these trends persevering throughout the experiments and that the primary finding of the paper relates to the different confidence attributions between human and AI counterparts, we suggest presenting this figure using the analysis from the following subsection (Confidence in humans versus machines, i.e., plotting the data by counterpart type). We understand this may be a trade-off between figure simplicity/readability, so I leave it to the authors discretion whether to enact that adjustment.

3. One interesting point that comes up is that people's assessments of machines is, to an extent, accurate and reflective of the performance of real AI systems. Machines tend to be poorly calibrated in terms of the posterior probabilities they assign during classification tasks, with the first-order response relatively loosely related to actual performance. Furthermore, the types of tasks that AI does poorly on also tends not to be the exact same problems that humans do poorly on, meaning that "difficulty" as defined relative to human participants may not have the same meaning. This might translate into expectations of overconfidence and attenuated relationships between difficulty and confidence for the machine decision-makers. This isn't really a "problem" with the paper, but rather a partial explanation of why the results may have turned out how they did – namely, that these expectations are (ecologically) valid in other domains.
a. For examples: Nourani, M., Kabir, S., Mohseni, S., & Ragan, E. D. (2019, October). The effects of meaningful and meaningless explanations on trust and perceived system accuracy in intelligent systems. In Proceedings of the AAAI Conference on Human Computation and Crowdsourcing (Vol. 7, pp. 97-105).
b. Zhang, Y., Liao, Q. V., & Bellamy, R. K. (2020). Effect of confidence and explanation on accuracy and trust calibration in AI-assisted decision making. In Proceedings of the 2020 conference on fairness, accountability, and transparency (pp. 295-305).

4. The analysis of learning effects across trials in experiments 1-3 demonstrated changes in confidence attribution following experience with the AI counterparts, especially when participants observed a human counterpart first. This finding has raised two points that we invite the authors to consider.

a. First, the authors suggest that the observed changes in confidence attributions are due to observing the AI counterpart's performance, but we offer the possibility that the feedback provided to participants regarding their own confidence estimates might have also driven that change. This is perhaps a minor point, but the implications between change via observation and change via explicit feedback might be worth consideration.
b. The second point also relates to the presence of participant feedback. That is, it is interesting that participants received feedback on their confidence attribution error and their estimates did NOT change. That is, confidence attributions to AI and human counterparts didn't converge to the underlying means of the confidence generation functions with error feedback. This is perhaps only noteworthy. In any case, we are not suggesting that these points detract from the authors' primary observation of these analyses: that people's confidence attributions to AI were certainly initially higher, indicating a prior belief that the AI would be more confident.

Minor comments / spelling:
- Small typo in the paragraph at the top of page 16: "altgough" -> "although".

Reviewer #4 (Remarks to the Author):

I co-reviewed this manuscript with one of the reviewers who provided the listed reports. This is part of the Communications Psychology initiative to facilitate training in peer review and to provide appropriate recognition for Early Career Researchers who co-review manuscripts.

* TRANSPARENT PEER REVIEW: Communications Psychology uses a transparent peer review system. This means that we publish the editorial decision letters including Reviewers' comments to the authors and the author rebuttal letters online as a supplementary peer review file. However, on author request, confidential information and data can be removed from the published reviewer reports and rebuttal letters prior to publication. If your manuscript has been previously reviewed at another journal, those Reviewers' comments would not form part of the published peer review file.

** Visit Nature Research's author and referees' website at <a

href="http://www.nature.com/authors">www.nature.com/authors for information about policies, services and author benefits**

Communications Psychology is committed to improving transparency in authorship. As part of our efforts in this direction, we are now requesting that all authors identified as 'corresponding author' create and link their Open Researcher and Contributor Identifier (ORCID) with their account on the Manuscript Tracking System prior to acceptance. ORCID helps the scientific community achieve unambiguous attribution of all scholarly contributions. You can create and link your ORCID from the home page of the Manuscript Tracking System by clicking on 'Modify my Springer Nature account' and following the instructions in the link below. Please also inform all co-authors that they can add their ORCIDs to their accounts and that they must do so prior to acceptance.
https://www.springernature.com/gp/researchers/orcid/orcid-for-nature-research

For more information please visit http://www.springernature.com/orcid

If you experience problems in linking your ORCID, please contact the Platform Support Helpdesk.

Version 1:

Decision Letter:

** Please ensure you delete the link to your author homepage in this e-mail if you wish to forward it to your coauthors **

Dear Dr Colombatto,

Your manuscript titled "Illusions of Confidence in Artificial Systems" has now been seen by our reviewers, whose comments appear below. In light of their advice I am delighted to say that we are happy, in principle, to publish a suitably revised version in Communications Psychology.

We therefore invite you to revise your paper one last time to address the remaining concerns of our reviewers and a list of editorial requests. At the same time we ask that you edit your manuscript to comply with our format requirements and to maximise the accessibility and therefore the impact of your work.

EDITORIAL REQUESTS:

Reporting and formatting of your manuscript are nearly fully aligned with our requirements. Thank you so much for implementing the requested changes at "revise". There are a few remaining issues. Please review our specific editorial comments and requests regarding your manuscript in the attached "Editorial Requests Table". Please outline your response to each request in the right hand column. Please upload the completed table with your manuscript files as a Related Manuscript file.

If you have any questions or concerns about any of our requests, please do not hesitate to contact me.

SUBMISSION INFORMATION:

In order to accept your paper, we require the files listed here https://www.nature.com/documents/commsj-file-checklist.pdf .

OPEN ACCESS:

Communications Psychology is a fully open access journal. Articles are made freely accessible on publication. For further information about article processing charges, open access funding, and advice and support from Nature Research, please visit https://www.nature.com/commspsychol/open-access

At acceptance, you will be provided with instructions for completing the open access licence agreement on behalf of all authors. This grants us the necessary permissions to publish your paper. Additionally, you will be asked to declare that all required third party permissions have been obtained, and to provide billing information in order to pay the article-processing charge (APC).

* TRANSPARENT PEER REVIEW: Communications Psychology uses a transparent peer review system. On author request, confidential information and data can be removed from the published reviewer reports and rebuttal letters prior to publication. If you are concerned about the release of confidential data, please let us know specifically what information you would like to have removed. Please note that we cannot incorporate redactions for any other reasons.

* CODE AVAILABILITY: All Communications Psychology manuscripts must include a section titled "Code Availability" at the end of the methods section. We require that the custom analysis code supporting your conclusions is made available in a publicly accessible repository at this stage; please choose a repository that generates a digital object identifier (DOI) for the code; the link to the repository and the DOI must be included in the Code Availability statement. Publication as

Supplementary Information will not suffice.

* DATA AVAILABILITY:
All Communications Psychology manuscripts must include a section titled "Data Availability" at the end of the Methods section. More information on this policy, is available in the Editorial Requests Table and at http://www.nature.com/authors/policies/data/data-availability-statements-data-citations.pdf.

Please use the following link to submit the above items:
Link Redacted
** This url links to your confidential home page and associated information about manuscripts you may have submitted or be reviewing for us. If you wish to forward this email to co-authors, please delete the link to your homepage first **

We hope to hear from you within two weeks; please let us know if you need more time.

Best regards,

Marike

Marike Schiffer, PhD
Chief Editor
Communications Psychology

REVIEWERS' COMMENTS:

Reviewer #1 (Remarks to the Author):

The authors have addressed my past comments and I am enthusiastic about the publication of this manuscript.

The revised introduction and discussion now offer a much clearer articulation of the theoretical context. The authors have expanded the discussion of the findings from Experiment 6, offering several plausible explanations for the lack of AI-human differences in the emotion categorization task. They have also included a table that provides a clear and concise summary of the key manipulations and findings across all seven experiments. And the revised discussion now includes an extended and meaningful section that explores the practical implications of the findings.

Reviewer #2 (Remarks to the Author):

I reviewed an initial submission of this work a few months ago. The authors provided a thorough and considerate response to my comments, together with corresponding changes in the manuscript. Since the first submission I was favourable to this work; I think the revision is stronger and I recommend acceptance.

I note that in some cases the authors basically responded to my comments by highlighting a need for further work -- this is fair enough (and of course any single study cannot exhaust all issues). I was a little bit sceptical of their response regarding my comment concerning conflation of process and accuracy: I do agree that accuracy sometimes becomes a proxy for assumptions about process, but I think in general process is a key element informing perceptions of accuracy; so without information about process, over smallish samples of decisions, it would be difficult to estimate confidence. But, I do not think it is worth belabouring the point further in your paper, I think your related new text is fine. The point that human agents might anthropomorphise, in a sense, artificial agents, by projecting human states onto them (such as confidence) is interesting; I do very much agree with you that we might use proxies for such states (e.g., in the case of confidence, reaction time). There is a complex set of possibilities here, e.g., ‘too fast’ might indicate sloppy decisions. I would have preferred a more systematic examination of how these artificial agents are described and how participants understand their operation, but it looks like this should a topic for a future study. I am happy with how you responded to my other comments.

Reviewer #3 (Remarks to the Author):

The authors have addressed our previous concerns with the manuscript through direct additions and changes to the text. We are now satisfied with the revised paper.

Reviewer #4 (Remarks to the Author):

I co-reviewed this manuscript with one of the reviewers who provided the listed reports. This is part of the Communications Psychology initiative to facilitate training in peer review and to provide appropriate recognition for Early Career Researchers who co-review manuscripts.

** Visit Nature Research's author and referees' website at www.nature.com/authors for information about policies, services and author benefits**

Open Access This Peer Review File is licensed under a Creative Commons Attribution 4.0 International License, which permits use, sharing, adaptation, distribution and reproduction in any medium or format, as long as you give appropriate credit to the original author(s) and the source, provide a link to the Creative Commons license, and indicate if changes were made.
In cases where reviewers are anonymous, credit should be given to 'Anonymous Referee' and the source.
The images or other third party material in this Peer Review File are included in the article's Creative Commons license, unless indicated otherwise in a credit line to the material. If material is not included in the article's Creative Commons license and your intended use is not permitted by statutory regulation or exceeds the permitted use, you will need to obtain permission directly from the copyright holder.
To view a copy of this license, visit https://creativecommons.org/licenses/by/4.0/

Responses to Reviewer #1

This paper investigates how humans infer confidence from other agents, and specifically how this process operates differently when the agent is artificial (AI) rather than human. Across seven pre-registered experiments, the authors demonstrate a consistent and systematic "illusion of confidence" in AI: human participants tend to attribute higher confidence to artificial agents compared to human ones, even when the two exhibit identical behavior.

I think this is an interesting and useful set of experiments. The experiments span a range of tasks including perceptual decision-making, general knowledge quizzes, emotion categorization, and advice-taking scenarios. This variety of tasks makes the findings robust and more interesting. Participants over-attribute confidence to AI across multiple contexts, and the effect is mediated by prior beliefs about AI competence. The authors propose that these results point to a fundamental cognitive bias that could have broad implications for human-AI collaboration and trust in automated systems.

I commend the authors for their pre-registration. The experiments are sufficiently powered, and have transparent exclusion criteria and statistical analyses. The manuscript is generally well written.

We were glad that the reviewer found our study interesting and our results robust!

I have a few main concerns/recommendations. First, I think the theoretical framework is lacking. The set of experiments are incremental and appear quite exploratory with little or no common framework regarding the factors influencing confidence judgments.

We thank the reviewer for the opportunity to elaborate on the theoretical framework of our study. In particular, we see now how much of our introduction focused on the human vs. AI distinction, while less background was provided on the other factors explored here as potential drivers of confidence judgments. To incorporate additional background on these factors, and how they may be related to the main agent manipulation, we have now expanded our introduction on p. 4:

> "On the one hand, there is reason to suspect that attributions of confidence may differ between humans and AI on the basis of agents' beliefs about their respective capabilities. For example, humans are typically more confident when they are more likely to be correct (i.e., they exhibit some degree of metacognitive calibration). As a result, a tendency to overestimate AI's capabilities [6-7] may result in inflated attributions of confidence for AI [39]. These same agent-specific properties also underlie the distinction between inferences of others' confidence and participants' own confidence in others' choices (e.g. [40-41]), since an advisor may be confident in responses that an observer knows to be inaccurate, revealing a discrepancy between other- and self-directed metacognition. But beyond general beliefs about agents' abilities, inferences about confidence should also depend on momentary assessments of individual decisions. A large body of work in perceptual metacognition has shown how the same agent can be more or less confident depending on properties of specific decisions (e.g., difficulty), as well as their own performance on individual trials (e.g., accuracy and response times). In particular, confidence is typically higher when the evidence is easier to discriminate (i.e., on easier trials) and the agent is more likely to be accurate [39]. This positive relationship between difficulty and

> confidence however is disrupted when agents make errors, where they instead report lower confidence on easier trials — where, presumably, they become aware that they have made an error [39, 42]. In summary, past work in perceptual metacognition has shown how confidence is shaped both by agent-specific properties and momentary decision properties, raising fundamental questions about the relative role of agent-specific beliefs and momentary observations in the attribution of confidence to other systems."

I find the connection to "metacognition" unclear or confusing. The "folk metacognition" as a way in which laypeople perceive and interpret confidence in others, including AI, is not well-founded on the factors investigated in the various experiments.

We agree this terminology may be confusing as confidence judgments capture but one facet of metacognitive processes, and so we have now revised the manuscript throughout to specifically refer to attributions of confidence rather than attributions of metacognition. For example, in the abstract, we now frame our research question as "how humans attribute confidence" and "attributions of confidence", rather than "metacognition." In addition, we have clarified the term 'folk metacognition' by defining this as "how the folk attributes to other agents metacognitive states such as confidence."

Also, related, the absence of an illusion in emotion categorization in Experiment 6 is intriguing, but the explanation could benefit from further elaboration. For instance, could participants simply perceive emotion judgments as more ambiguous, reducing confidence judgments overall?

Thank you for highlighting this point: we agree that the description of those results could benefit from further explanation. We have now added a new paragraph in the discussion, where we elaborate on potential explanations for this result, including a role of overall decisional ambiguity as suggested by this reviewer. As we write on p. 32:

> "The impact of prior beliefs about accuracy demonstrated in the causal manipulation in Experiment 7 may also explain why illusions of confidence were strongest in tasks where AI is presumably highly accurate (e.g., visual detection in Experiments 1-3), and were minimised in more subjective tasks (e.g., emotion categorisation in Experiment 6). In particular, participants may have a prior belief that AI systems may be less accurate at emotion recognition given the common intuition that machines, unlike humans, do not themselves have the capacity to experience emotion [76]. We note, however, that a prior belief that humans may be more accurate in this task did not result in a reversal of the illusion, as observers did not perceive humans to be more confident than AI. This suggests that both performance priors and a general bias toward perceiving AI as more confident likely work together to produce the observed effect. Alternatively, the more subjective nature of the task may have led observers to rely on immediate behavioural cues (e.g., RTs) that were identical across agent types, rather than a priori beliefs about agents' accuracy, resulting in no statistically significant differences between agent types. Confidence about subjective quantities may also rely more on assessments of choice consistency than objective correctness [77-78]. Finally, the lack of a reliable difference between human and AI confidence in this domain may also reflect properties of the task itself; for example, participants may be attributing overall lower confidence to all agents given the more ambiguous nature of the task, resulting in a floor effect. This possibility

> seems unlikely, given that mean confidence ratings were comparable across experiments, and if anything, higher in emotion categorisation (average Exp. 1=53.22; Exp. 2=61.30; Exp. 3=57.29; Exp. 4=62.20; Exp. 6=70.15). Rather than changes in overall confidence, we thus interpret these findings as reflecting either different ability priors, such that AI systems are no longer believed to be more accurate, or a shift in cue weighting, where observers cease to rely on prior beliefs and rather base confidence attributions on directly observable behavioural indicators (e.g., reaction time and choice consistency) that did not differ across agents."

Second, just like the authors present some common figures across experiments, a summary table reporting the main results and statistics across experiments would be useful. It is very easy to get lost in reading with the multiple numbers and tests in-between.

Thank you for this suggestion! We agree a summary table would be helpful in summarising the results across experiments, and we have now added this on p. 17:

Exp	N	Task	H1: Decision Variables			H2: Agent Type
			Difficulty	Accuracy	RTs	
1	120	Visual Perception	✓	✓	✓	✓
			(interaction) ✓			AI > human
2	120	Visual Perception	✓	✓	N/A	✓
			✓			AI > human
3	60	Visual Perception	✓	✓	N/A	✓
			✓			AI > human
4	60	General Knowledge	✓	N/A	✓	✓
			✓			AI > human
5	60	Visual Perception	✓	✓	N/A	✓
			✓			AI > human
6	60	Emotion Perception	N/A	✓	✓	✗
7	60	Visual Perception	✓	✓	N/A	✓
			✓			High > low

Table 1. Summary of experimental designs and key findings. The table lists each experiment (Exp), sample size (N), task domain, and primary effects on confidence attributions. Columns indicate whether confidence was significantly predicted by decision variables (H1: difficulty, accuracy, reaction time) and by agent type (H2: human vs. AI), highlighting how the influence of these factors varied across tasks. RTs = Response times; ✓ = $p<.05$; ✗ = $p>.05$.

Finally, I recommend that the authors expand on the implications of these findings for AI design. How do these findings translate into real-world systems? I would like to see a more concrete proposal for mitigating the illusion of confidence.

Thank you for this suggestion; we agreed that a discussion of the implications for system design would be helpful in clarifying the impact and relevance of these findings, and we now discuss these in a new section of our discussion, on p. 34:

> "Given the role of confidence in regulating interpersonal trust and decision-making, our findings of an illusion of confidence in AI systems highlight the necessity for developing solutions for artificial metacognition that allow AI agents to appropriately communicate (potentially low) degrees of confidence in their behaviour [67]. Future work could explore types of confidence broadcasts (e.g., explicit numerical estimates, or implicit visual indicators) to facilitate alignment in human-AI interactions, and how these might help counter inaccurate priors [36]. Beyond highlighting the importance of integrating confidence estimates in AI systems, the studies reported here reveal specific determinants of confidence attributions, including prior beliefs and decision speed, which are likely to mislead observers and could be targeted in AI design to prevent erroneous attributions. For instance, the results of accuracy priors in Experiment 7 suggest that inflated attributions of confidence can be prevented by communicating the overall accuracy of the system. Similarly, the systems could automatically detect cases where users are likely to infer high confidence (e.g., on fast deliberation times), to flag potential divergences between objective accuracy and users' perceptions of performance."

Responses to Reviewer #2

I enjoyed reading this paper on confidence attributions. Naïve participants observed the behaviour of (assumed, as per experimenter instructions) either human or artificial agents, the latter corresponding to robots, neural networks, or algorithms. The observed behaviour concerned a perceptual task (matching noise patterns to a Gabor patch) or a general knowledge task (questions concerning country population) or an emotional categorisation task. Behaviour was matched between human and artificial agents. Note, in most experiments the target DV was judgments of how confident the agent (human or artificial) would be in their judgments; in one case, there was a DV of whether the participant would take advice from an agent (the participant would make a judgment and then given the opportunity to revise the judgment, based on agent feedback). There were several checks that the experiments worked sensibly, for example, confidence attributions went hand in hand with lower RT and higher accuracy. The main research question of the researchers is whether confidence attributions would vary between (assumed) human and artificial agents. The main finding is that, fairly uniformly, artificial agents would be considered as having more confidence in their responses, compared to human agents. There was one exception, namely there was no difference in confidence attributions, when the task was one of emotional categorisation.

There are many positives in this work. The overall result is interesting. The methodology is very meticulous and the range of manipulations extensive and convincing. I think this work will interest (and excite) many readers. Given the careful and rigorous methodology, I think most readers will accept the presented conclusions. I do have some objections – these do not attest to the quality of the research, rather to the way I interpreted the results. I do not intend these objections to be seen as undermining publication prospects; I rather offer them as indications of the kind of debate that this paper is likely to generate.

We were glad that the reviewer found our work interesting and the methodology sound, and we thank them for their helpful suggestions and constructive feedback.

Foremostly, the authors label their result as an illusion of confidence – that is, an illusion that an AI agent would be more confident in their decisions than a corresponding human agent, even if observed behaviour between the two kinds of agents is identical. As an aside, did participants notice / comment on the similarities between ostensibly human and artificial agents?

While our studies did not include a specific question probing participants' impressions of the similarities between agents, we did examine participants' responses to an open-ended question at the end of the study, asking them whether they had "notice[d] anything interesting during the experiment [they] wanted to share." Most participants remarked on their own performance, e.g. noting how the task became increasingly difficult over the course of the study (which was expected, as difficulty was adjusted over the course of the first part via a staircasing procedure). Few remarked on the differences between agents, and there was no particular pattern of comments regarding the similarities between agents. For example, a couple of participants in the experiments involving visual perception tasks (Experiments 1-3, 5) remarked on how the human agent seemed to be more accurate, while others noted how the robot seemed more accurate. Some participants explicitly mentioned relying on observed response times to infer the agents' confidence, but with no mention of differences between agents. We note however that this question was rather open-ended, and more detailed debriefing may have revealed additional patterns — a possibility we will keep in mind for future work.

In any case, I could not agree with the label 'illusions'. I think confidence in a decision / judgment is a function of many factors: one of the factors is content, so that for very simple decisions, one can imagine that the generating agent would be confident. However, another key factor, and one that does not seem to be acknowledged in this work, is the process via which an answer is generated. For example, if we consider a long division, e.g., 5345633 divided by 3452, both a human and an artificial agent (at the simplest level, a calculator!) may arrive to the same answer. However, would we not have more faith in the artificial agent, on the back of assumptions that artificial agents are carrying out this process on the basis of multiply verified and logically correct algorithmic processes? Likewise, say, in considering an ailing patient, both a naïve observer and an expert doctor might offer the same advice, but would it not be the case that we would attribute more confidence to the doctor?

Thank you for raising this important point regarding potential sources of confidence attributions. We agree that attributions of confidence may reflect not only the content of a decision, but also beliefs about the processes by which a decision is reached. In fact, the differences we report between humans and AI agents may reflect exactly these sorts of inferences about underlying decision processes. At the same time, we believe these underlying processes may ultimately influence perceived confidence because they shape perceptions of likely reliability and accuracy. In other words, decision processes matter for confidence attributions because they result in outcomes that are more or less likely to be correct, rather than by virtue of specific properties of the processes themselves. For instance, in the example this reviewer mentions, it is unclear whether higher attributions of confidence to calculators would result from the specific nature of "algorithmic processes" or the fact that they are "multiply verified and logically correct", and thus more likely to be accurate.

Similarly, it is unclear whether higher attributions of confidence to expert doctors would result from specific features of their decision-making process (e.g., deductive reasoning) or the fact that these particular processes are more likely to be accurate than a naïve observer. We thus suspect that this reviewer's intuition about the role of decision processes in attributions of confidence may be captured by prior beliefs about accuracy, rather than particular features of the decisions themselves. In this context, the term 'illusions' seems apt, given that higher attributions of confidence were rooted in prior beliefs about accuracy that were not reflected in the evidence available to observers, which consisted of identical behaviour. A useful analogy here is with perceptual "illusions" such as Adelson's checkerboard, in which the contrasts of two identical image patches are perceived differently, presumably due to low-level priors about the influence of lighting on surface reflectance. It remains however possible that specific features of decision processes may govern attributions of confidence independent of their likely accuracy, a possibility we now acknowledge on p. 31:

> "It also remains possible that, in addition to overall beliefs about accuracy, differential attributions of confidence to humans and AI systems may have resulted from observers' beliefs about their different decision processes (e.g., AI's algorithmic or logical versus humans' intuitive and probabilistic reasoning). While we suspect that, ultimately, assumptions about decision processes may impact attributed confidence to the extent that they impact accuracy (e.g., an answer from logic being more likely to be accurate than one from intuition), future work may examine the relative contribution of these factors by varying properties of the internal functionality of the system independent of its performance output [75]."

Another related issue is that equating performance between human and artificial agents would very much be a function of what kind artificial agent we are talking about. As a trivial example, a calculator is extremely capable in arithmetic but useless in language tasks. Therefore, the importance of equating behaviour between artificial and human agents is suspect in this way too; the only way I could see of addressing this issue is through more detail of the kind of artificial agent relevant in each experiment (more so than is currently provided, noting that, for example, for an average naïve participant algorithm or neural networks may not mean much).

We entirely agree that the type of artificial agent considered is critical, since confidence attributions may largely depend on the specific capacities of the agent. This is in part why we aimed to replicate the key findings of our manuscript across different types of artificial agents, from a more anthropomorphic robot in Experiments 1-3, to a computer algorithm described to participants as having been "built and trained to complete this task" (Experiments 4-7). Of course, this was mostly intended to be a demonstration of robustness across different agent framings, rather than a systematic investigation of agent descriptions and capacities, and we recognize that participants' interpretations of such labels (e.g., "robot", "algorithm") may vary widely, with different implications for attributions of confidence. Similar to the case of decision processes discussed above, however, we believe that much of this variation ultimately reflects differences in the perceived abilities of the agent. The possibilities outlined by this reviewer are thus consistent with the mechanistic explanation we hypothesize and test in Experiment 7, which highlights a role for beliefs about an agent's accuracy on a task (akin to this reviewer's example of "a calculator [being] extremely capable in arithmetic but useless

in language tasks"). We now highlight and further elaborate on the potential role of agent type on p. 35, where we write that:

> "[T]he studies reported here tested illusions of confidence across different agent descriptions, from robots (Experiments 1-3) to computer algorithms (Experiments 4-7), but AI systems can vary widely in terms of their functions and appearance. Future work may further examine how participants interpret these labels and potential assumptions they might have about different types of artificial agents, particularly with respect to their capacities and abilities, which in turn would influence attributions of confidence."

Another issue is that the main DV – attributions of how confident an agent is – struck me as a little bit odd, in that it assumes that both human and artificial agents share a similar mechanism of self-reflection and self-evaluation. In some cases, this is clearly less meaningful: for example, we could not ask whether a calculator is confident in the response it provides for long division. Some care is needed here, because there is a danger that, in some respects, the research question could be criticised as suspect. Note, I am curious as to why the authors did not consider instead the question of trust in human vs AI agents. This is done in one of the experiments, where participants were asked to decide whether to revise their response, but it is certainly not one of the main questions. I do appreciate that what I am interested in is not necessarily what the authors are interested in; however, as per the above, I do think that there is a need to explain what it means (what the authors think it means) for an artificial agent to be confident or not in a decision (and so what attributions participants are making or think they are making).

Thank you for raising this important point. We entirely agree with this reviewer that asking "how confident" an agent seems to be presupposes that observers attribute some capacity for metacognition to the agent. In some cases, such as the calculator mentioned here, this clearly does not apply, since most participants would deny that calculators have the capacity for self-awareness. When it comes to artificial intelligence as tested in the current studies, this seems more ambiguous, as their capacities are still developing, and there is substantial debate on their capacities even among experts. The key consideration that inspired the current studies, however, is that regardless of whether observers are *explicitly* attributing the capacity for self-awareness to AI systems, the way in which their output is communicated still involves cues that, in humans, are *implicitly* associated with confidence. This raises the possibility that observers may attribute confidence to specific decisions, above and beyond overall judgments of these systems' abilities. We have now clarified this on pp. 3-4, where we write that:

> "[L]little is currently known about how humans attribute confidence to AI systems. In fact, this type of inference is difficult when interacting with machines which are typically designed to fulfil first-order rather than metacognitive functions, and which vary in the extent to which they can provide calibrated estimates of uncertainty. Indeed, AI's capacity for metacognition, including the ability to provide accurate confidence reports, is developing […] However, the format of their output often contains cues that, in humans, typically do signal confidence implicitly, such as system response times which are akin to human response times. Together, these considerations raise foundational questions around how humans attribute confidence to AI systems when these are not communicated explicitly."

We also note that, with regard to trust, we entirely share the reviewer's interest; in fact, part of the motivation for studying attributions of confidence is that these play a key role in

regulating group decision-making and delegation in humans. But while trust is a multifaceted social evaluation of an agent's reliability, attributions of confidence concern a more immediate inference about the agent's epistemic state in a specific decision context. The latter thus offers a more granular window into how users interpret AI output during tasks, which may in turn fuel more general impressions of trust. This link was also evident in each of the experiments we report, where attributions of confidence are positively related to the perceived trustworthiness of the systems. As we note on p. 31,

"Higher attributions of confidence may, in turn, reinforce the perceived accuracy of the responses, creating a feedback loop that amplifies both trust in the system and in the suggestions [57]"; and while "future work is needed to probe possible causal relationships between perceived confidence and social impressions, this result suggests that illusions of confidence may play an important role in regulating trust in interactions."

Minor methods issues: the analyses on changes with time are interesting, but I was unsure what to make of them. It is well-known that there are question order effects or evaluation biases (that is, biases from considering some earlier information and responding to it). It is possible that some readers might feel that the analyses are a little superfluous. Perhaps either explain better why you are doing them or move them to supplementary material?

Thank you for this suggestion. We also see these analyses as secondary to the main effect of interest (i.e., the overall difference between humans and AI), but they do provide some insight into the mechanisms underlying these, particularly with respect to the relative contribution of prior beliefs over observed performance. In this sense, the finding that illusions of confidence are strongest at the beginning of the relevant blocks provides further evidence for the mechanistic explanation we propose regarding a causal role for prior beliefs about accuracy. We have thus retained them in the main text, while also highlighting their significance in the context of our study, on p. 31:

> "Indeed, further analyses of learning effects revealed that this bias was stronger at the beginning of the experimental blocks, and diminished thereafter as participants observed the agents' behaviour. This updating may in part reflect the feedback on the accuracy of their confidence attributions that was provided to participants every few trials, which participants may have used to guide their estimates. However, we consider this unlikely, as the feedback was provided as a summary statistic (i.e., a cumulative points total) and was non-directional (i.e., both overestimation and underestimation of confidence would yield the same feedback outcome). In addition, despite a reduction in its magnitude, the illusion of confidence remained robust toward the end of the experiment, suggesting that it was relatively resistant to feedback. Instead, we suggest that this reduction reflects a discrepancy between participants' prior beliefs about an agent (e.g., that the algorithm would excel in general knowledge) and its behaviour (which was sometimes inaccurate). This was further confirmed by an experimental manipulation of prior beliefs about performance in Experiment 7 which demonstrated that identical decisions were perceived as more confident when participants held a prior belief that the algorithm was more accurate."

Also, with the mixed effects analyses, it would be helpful to explain more clearly whether you employed slopes and if yes for which effects; and, if you did employ slopes, what kind of covariance matrix you assumed.

Random effects in the preregistered models were specified as intercepts, not slopes; we have now checked that this is clearly stated in the Statistical Analysis section of the Methods, e.g. "we conducted linear mixed-effects models of the effect of task difficulty […], accuracy […], and response times […] on the confidence estimates, with counterpart participant […] and observer number as random intercepts."

Responses to Reviewer #3

The paper presents seven pre-registered studies investigating human attributions of confidence to artificially intelligent systems (AI) in comparison to performance-matched human counterparts. The authors' primary finding is that people ascribed higher confidence to the decisions of AI agents than humans. This effect is demonstrated across agent descriptions and various objective perceptual tasks, is reduced under a subjective emotional identification task, and is shown to be influenced by prior beliefs about AI capabilities.
We found this manuscript to be a comprehensive and well-executed study that addresses an important, relevant, and timely area of research in human-AI interaction. Overall, the manuscript is clear and well-written. It presents a robust set of experiments that contribute meaningfully to our understanding of human perception of AI's metacognitive states. We have a few comments on the interpretation of specific experimental outcomes and a suggestion on the data used in a figure.

We are grateful to these reviewers for their assessment of our work and for their helpful suggestions.

Major suggestions:
1. Generalizability beyond matched performance: The study's design matches the performance of the human and AI counterparts to the performance of the observer/participant. This is a nicely designed control to examine the phenomena across these several studies. However, it raises a question about the generalizability or the extent of the illusion of confidence when humans interact with human and AI counterparts whose performance levels are notably different from their own (better OR worse). Many of the nuanced findings in the literature around human-AI trust are often moderated by such nuanced relationships. We suggest that the discussion section considers the implications of this design choice for the broader applicability of this finding.

Thank you for raising this point. We entirely agree that both human-human and human-AI interactions often involve differing levels of ability, and indeed, these are precisely the contexts in which expressions of confidence are most important, as they help decision makers weigh competing opinions according to their likely accuracy. While matching agents' performance to that of the observers in the present studies allowed us to isolate attributions of confidence from objective performance differences, the reviewers are right to point out that this design leaves open questions about situations involving unequal ability. On the one hand, the findings of Experiment 7 already point to a clear role for perceived agent skill, with

inferences of accuracy being higher for agents believed to be more competent. But beyond the agent's actual ability, we agree that the magnitude and direction of confidence attributions are likely to also depend on the observer's own performance. For instance, we speculate that more skilled observers may attribute lower confidence to others because they can better detect inaccuracies, whereas less skilled observers may over-attribute confidence when they cannot discriminate errors. We now note this design choice and its implications on p. 35, where we write that:

> "Second, in all current studies the agents' accuracy in the inference task was matched to that of the observers. While this design choice allowed us to isolate attributions of confidence from objective performance differences, future work should examine conditions in which agents display higher or lower ability than the observer. The present results already highlight a critical role of perceived agent accuracy, both at the trial level (as reflected in the main effects of accuracy that emerged in all experiments), and at the global level (as demonstrated by the causal manipulation of accuracy in Experiment 7). We further speculate that these effects may interact with observers' own ability, such that more skilled observers might attribute lower confidence to others because they are better able to detect inaccuracies, whereas less skilled observers might over-attribute confidence to others when they cannot reliably discriminate errors."

2. Following from the previous point - Figure 2 demonstrates the nice effects of changes to perceived confidence (A) for RT and (B) task difficulty by correct and incorrect responses and captures an intuitive finding for the relationship between confidence attributions and RT/accuracy. Given that the authors report these trends persevering throughout the experiments and that the primary finding of the paper relates to the different confidence attributions between human and AI counterparts, we suggest presenting this figure using the analysis from the following subsection (Confidence in humans versus machines, i.e., plotting the data by counterpart type). We understand this may be a trade-off between figure simplicity/readability, so I leave it to the authors discretion whether to enact that adjustment.

Thank you for this suggestion! This figure has now been updated to show the effects separately for each agent type.

3. One interesting point that comes up is that people's assessments of machines is, to an extent, accurate and reflective of the performance of real AI systems. Machines tend to be poorly calibrated in terms of the posterior probabilities they assign during classification tasks, with the first-order response relatively loosely related to actual performance. Furthermore, the types of tasks that AI does poorly on also tends not to be the exact same problems that humans do poorly on, meaning that "difficulty" as defined relative to human participants may not have the same meaning. This might translate into expectations of overconfidence and attenuated relationships between difficulty and confidence for the machine decision-makers. This isn't really a "problem" with the paper, but rather a partial explanation of why the results may have turned out how they did – namely, that these expectations are (ecologically) valid in other domains.
a. For examples: Nourani, M., Kabir, S., Mohseni, S., & Ragan, E. D. (2019, October). The effects of meaningful and meaningless explanations on trust and perceived system accuracy in intelligent systems. In Proceedings of the AAAI Conference on Human Computation and Crowdsourcing (Vol. 7, pp. 97-105).
b. Zhang, Y., Liao, Q. V., & Bellamy, R. K. (2020). Effect of confidence and explanation on accuracy and trust calibration in AI-assisted decision making. In Proceedings of the 2020 conference on fairness, accountability, and transparency (pp. 295-305).

We entirely agree that that these accuracy priors may be relative to human performance, and in fact, as noted in response to a similar point raised by Reviewer 2, these are precisely the contexts in which confidence is most useful. We now explicitly discuss this point in our General Discussion on p. 35, where we write that

> "The present results already highlight a critical role of perceived agent accuracy, both at the trial level (as reflected in the main effects of accuracy that emerged in all experiments), and at the global level (as demonstrated by the causal manipulation of accuracy in Experiment 7). We further speculate that these effects may interact with observers' own ability, such that more skilled observers might attribute lower confidence to others because they are better able to detect inaccuracies, whereas less skilled observers might over-attribute confidence to others when they cannot reliably discriminate errors."

4. The analysis of learning effects across trials in experiments 1-3 demonstrated changes in confidence attribution following experience with the AI counterparts, especially when participants observed a human counterpart first. This finding has raised two points that we invite the authors to consider.

a. First, the authors suggest that the observed changes in confidence attributions are due to observing the AI counterpart's performance, but we offer the possibility that the feedback provided to participants regarding their own confidence estimates might have also driven that change. This is perhaps a minor point, but the implications between change via observation and change via explicit feedback might be worth consideration.

This sounds right to us, and we now explicitly note this possibility in the Discussion, on pp. 31:

"This updating may in part reflect the feedback on the accuracy of their confidence attributions that was provided to participants every few trials, which participants may have used to guide their estimates. However, we consider this unlikely, as the feedback was provided as a summary statistic (i.e., a cumulative points total) and was non-directional (i.e., both overestimation and underestimation of confidence would yield the same feedback outcome)."

b. The second point also relates to the presence of participant feedback. That is, it is interesting that participants received feedback on their confidence attribution error and their estimates did NOT change. That is, confidence attributions to AI and human counterparts didn't converge to the underlying means of the confidence generation functions with error feedback. This is perhaps only noteworthy. In any case, we are not suggesting that these points detract from the authors' primary observation of these analyses: that people's confidence attributions to AI were certainly initially higher, indicating a prior belief that the AI would be more confident.

This also sounds right to us. We suspect that this 'failure' of updating may be due in part to the format of the feedback, which was too coarse (i.e., infrequent) and vague (i.e., non-directional) to support systematic updating. Alternatively, this stability may indicate that participants relied on relatively stable priors about each agent's confidence that were resistant to change even in the presence of limited feedback. This point is now also noted on p. 31: "despite a reduction in its magnitude, the illusion of confidence remained robust toward the end of the experiment, suggesting that it was relatively resistant to feedback."

Minor comments / spelling:
- Small typo in the paragraph at the top of page 16: "altgough" -> "although".

Fixed, thank you!

Response to Reviewer #4

I co-reviewed this manuscript with one of the reviewers who provided the listed reports. This is part of the Communications Psychology initiative to facilitate training in peer review and to provide appropriate recognition for Early Career Researchers who co-review manuscripts.

Thank you for your thoughtful review!
