# Supplementary material for: Beliefs about accuracy shape confidence attributions to humans and artificial agents
Source: Commun Psychol. 2026 Apr 22;4:114. doi: 10.1038/s44271-026-00445-4 (PMC13461344; doi:10.1038/s44271-026-00445-4)
Supplement: Supplementary file 2 [file 44271_2026_445_MOESM2_ESM.pdf]

nature portfolio

Corresponding author(s): Clara Colombatto

Last updated by author(s): Jun 11, 2025

Reporting Summary

Nature Portfolio wishes to improve the reproducibility of the work that we publish. This form provides structure for consistency and transparency in reporting. For further information on Nature Portfolio policies, see our Editorial Policies and the Editorial Policy Checklist.

Statistics

For all statistical analyses, confirm that the following items are present in the figure legend, table legend, main text, or Methods section.

n/a	Confirmed	
☐	☒	The exact sample size (n) for each experimental group/condition, given as a discrete number and unit of measurement
☐	☒	A statement on whether measurements were taken from distinct samples or whether the same sample was measured repeatedly
☐	☒	The statistical test(s) used AND whether they are one- or two-sided<br>*Only common tests should be described solely by name; describe more complex techniques in the Methods section.*
☐	☒	A description of all covariates tested
☐	☒	A description of any assumptions or corrections, such as tests of normality and adjustment for multiple comparisons
☐	☒	A full description of the statistical parameters including central tendency (e.g. means) or other basic estimates (e.g. regression coefficient) AND variation (e.g. standard deviation) or associated estimates of uncertainty (e.g. confidence intervals)
☐	☒	For null hypothesis testing, the test statistic (e.g. F, t, r) with confidence intervals, effect sizes, degrees of freedom and P value noted<br>*Give P values as exact values whenever suitable.*
☒	☐	For Bayesian analysis, information on the choice of priors and Markov chain Monte Carlo settings
☒	☐	For hierarchical and complex designs, identification of the appropriate level for tests and full reporting of outcomes
☐	☒	Estimates of effect sizes (e.g. Cohen's d, Pearson's r), indicating how they were calculated

Our web collection on statistics for biologists contains articles on many of the points above.

Software and code

Policy information about availability of computer code

Data collection	Stimulus presentation and data collection were controlled via custom software written in HTML, CSS, JavaScript, and PHP, using the jsPsych library, version 7.3.0, and the Jatos Mindprobe server.
Data analysis	Analyses were conducted using R version 4.2.1, RStudio version 2022.07.2, and the following packages: dplyr version 1.0.10, tidyr version 1.3.0, kableExtra version 1.3.4, ggplot2 version 3.4.0, lme4 version 1.1.31, lmerTest version 3.1.3, emmeans version 1.8.2, effectsize version 0.8.2. Metacognitive efficiency scores were computed in Matlab version 2022a using JAGS version 3.4.0 and the HMeta-d package version 0.2 (https://github.com/metacoglab/HMeta-d).

For manuscripts utilizing custom algorithms or software that are central to the research but not yet described in published literature, software must be made available to editors and reviewers. We strongly encourage code deposition in a community repository (e.g. GitHub). See the Nature Portfolio guidelines for submitting code & software for further information.

April 2023

Data

Policy information about availability of data

All manuscripts must include a data availability statement. This statement should provide the following information, where applicable:

- Accession codes, unique identifiers, or web links for publicly available datasets
- A description of any restrictions on data availability
- For clinical datasets or third party data, please ensure that the statement adheres to our policy

Anonymised raw data for all experiments are openly available on the Open Science Framework (OSF) website at this link: https://osf.io/8tphs/.

Research involving human participants, their data, or biological material

Policy information about studies with human participants or human data. See also policy information about sex, gender (identity/presentation), and sexual orientation and race, ethnicity and racism.

Reporting on sex and gender	Participants were not specifically sampled based on sex or gender, and we do not perform any sex- or gender-specific analysis. However, gender data were collected to provide a group-level description of the sample, with a self-report question in the debrief stage of the experiment ("What gender do you identify with?", to be answered with open-ended text).
Reporting on race, ethnicity, or other socially relevant groupings	Data on race, ethnicity, or other socially relevant groupings were not collected as these were not relevant to our hypotheses.
Population characteristics	See below ("Behavioural & social sciences study design").
Recruitment	Participants were recruited via Prolific (prolific.com) in exchange for monetary compensation, and qualified for the study if they (1) were fluent in English; (2) had a Prolific task approval rate of at least 95%; and (3) had not previously participated in any of the other studies reported in the manuscript. Participants self-enroll into Prolific, and it is thus possible that this sample may differ from the general population; however, this self-selection is unlikely to impact the findings of the current manuscript.
Ethics oversight	All methods and procedures were approved by the UCL Research Ethics Committee (Project ID Number: 1260/003). Informed consent was obtained from all participants.

Note that full information on the approval of the study protocol must also be provided in the manuscript.

Field-specific reporting

Please select the one below that is the best fit for your research. If you are not sure, read the appropriate sections before making your selection.

☐ Life sciences ☒ Behavioural & social sciences ☐ Ecological, evolutionary & environmental sciences

For a reference copy of the document with all sections, see nature.com/documents/nr-reporting-summary-flat.pdf

Behavioural & social sciences study design

All studies must disclose on these points even when the disclosure is negative.

Study description	All experiments involved quantitative experimental designs.
Research sample	We recruited samples of adults from Prolific (prolific.com), an online crowdsourcing marketplace. A total of N=120 participants completed Experiments 1 and 2, and a total of N=60 completed Experiments 3 to 7. The final samples for each experiment were N=113 in Experiment 1 (26 females, Mage=31.65), N=109 in Experiment 2 (32 females, Mage=33.76), N=56 in Experiment 3 (25 females, Mage=33.07), N=59 in Experiment 4 (24 females, Mage=33.22), N=52 in Experiment 5 (21 females, Mage=33.71), N=60 in Experiment 6 (25 females, Mage=31.12), and N=57 in Experiment 7 (21 females, Mage=30.82).
Sampling strategy	These experiments employed a voluntary response sampling procedure from the Prolific participant pool. The sample size for Experiment 1 was determined via a power analysis of pilot data (N=18) and was fixed for Experiment 2. The sample size for the remaining experiments was halved to N=60 based on a power analysis of Experiments 1 and 2.
Data collection	Participants completed the experiment online on their own devices.
Timing	Experiment 1: 2022/12/21 - 2023/01/11<br>Experiment 2: 2023/02/09 - 2023/02/15<br>Experiment 3: 2023/03/08 - 2023/03/10<br>Experiment 4: 2023/03/22 - 2023/03/24<br>Experiment 5: 2023/04/04 - 2023/04/26

	Experiment 6: 2024/04/16 - 2024/04/19<br>Experiment 7: 2024/09/15 - 2023/10/03
Data exclusions	Participants were excluded according to our pre-registered plans if they (1) had a viewport size smaller than 720x500px (N=1 in Experiment 4); (2) reported having encountered problems (see Debriefing; N=2 in Experiment 1, N=1 in Experiment 2); (3) failed to answer our open-ended text questions sensibly (see Debriefing; N=1 in Experiment 1, N=1 in Experiment 5); (4) had a mean accuracy lower than 70% in the catch questions about the agents' confidence in Experiment 5 (N=6), and about the agents' accuracy in Phase 1 of Experiment 7 (N=17); (5) converged on a target with opacity level lower than 5% in Phase 1 (N=4, 10, 4, 1; in Experiments 1, 2, 3, and 5, respectively); and (6) provided uniform confidence ratings in Phase Two (i.e. with 80% of ratings below 10% or above 90%; N=3 in Experiment 7). No participants participated more than once as determined via their Prolific IDs.
Non-participation	Participants who declined consent or did not complete the study were not included in the dataset.
Randomization	Participants were not allocated into experimental groups. Some experimental variables were randomly determined for each participant (e.g., the order of blocks) via the Math.random() function in Javascript.

Reporting for specific materials, systems and methods

We require information from authors about some types of materials, experimental systems and methods used in many studies. Here, indicate whether each material, system or method listed is relevant to your study. If you are not sure if a list item applies to your research, read the appropriate section before selecting a response.

Materials & experimental systems

n/a	Involved in the study
☒	☐ Antibodies
☒	☐ Eukaryotic cell lines
☒	☐ Palaeontology and archaeology
☒	☐ Animals and other organisms
☒	☐ Clinical data
☒	☐ Dual use research of concern
☒	☐ Plants

Methods

n/a	Involved in the study
☒	☐ ChIP-seq
☒	☐ Flow cytometry
☒	☐ MRI-based neuroimaging

Plants

Seed stocks	*Report on the source of all seed stocks or other plant material used. If applicable, state the seed stock centre and catalogue number. If plant specimens were collected from the field, describe the collection location, date and sampling procedures.*
Novel plant genotypes	*Describe the methods by which all novel plant genotypes were produced. This includes those generated by transgenic approaches, gene editing, chemical/radiation-based mutagenesis and hybridization. For transgenic lines, describe the transformation method, the number of independent lines analyzed and the generation upon which experiments were performed. For gene-edited lines, describe the editor used, the endogenous sequence targeted for editing, the targeting guide RNA sequence (if applicable) and how the editor was applied.*
Authentication	*Describe any authentication procedures for each seed stock used or novel genotype generated. Describe any experiments used to assess the effect of a mutation and, where applicable, how potential secondary effects (e.g. second site T-DNA insertions, mosiacism, off-target gene editing) were examined.*
